# Trefoil Factor 3 Inhibits Thyroid Cancer Cell Progression Related to IL-6/JAK/STAT3 Signaling Pathway

**DOI:** 10.1155/2021/2130229

**Published:** 2021-09-14

**Authors:** Yunchao Xin, Xiaoling Shang, Xiaoran Sun, Yachao Liu, Guogang Xu, Gang Xue

**Affiliations:** ^1^Department of Otolaryngology Head and Neck Surgery, The First Affiliated Hospital of Hebei North University, Zhangjiakou, Hebei 075000, China; ^2^Department of Gastroenterology, The First Affiliated Hospital of Hebei North University, Zhangjiakou, Hebei 075000, China

## Abstract

**Objectives:**

Abnormal expression of trefoil factor 3 (TFF3) in breast, stomach, and colon tumors may be related to the occurrence of tumors, suggesting its role in angiogenesis. In this study, the aim was to explore the role of TFF3 in thyroid cancer.

**Methods:**

TFF3 expression analysis was performed via GEPIA and RT-PCR. To explore the effects of TFF3 on thyroid cancer cell motility, cell function assays were performed. Furthermore, GSEA pathway analysis and western blot were used to explore the mechanism by which TFF3 represses the progression of thyroid cancer cells.

**Results:**

Here, we showed that low expression level of TFF3 in thyroid cancer is related to thyroid cancer nodal metastasis. The patients with low TFF3 expression showed worse disease-free survival than those with high level of TFF3. Underexpressed TFF3 increased cell motility and inhibited cell apoptosis. We found that the levels of IL-6, p-JAK2/JAK2, and pSTAT3/STAT3 were inhibited in the pcDNA-TFF3 group compared to the pcDNA-NC group and these factors were upregulated in the si-TFF3 group compared to the si-NC group in BCPAP and TPC-1 cells.

**Conclusion:**

TFF3 inhibits thyroid cancer cell progression related to IL-6/JAK/STAT3 signaling pathway.

## 1. Introduction

The statistical incidence of thyroid cancer has shown a younger trend in recent years, and the cervical lymph node metastasis rate can be as high as 23%–56% [1]. Although most differentiated thyroid cancers can be surgically removed, postoperative adjuvant radiation therapy can obtain good results. However, 10%–20% of patients still suffer from dedifferentiation. Such thyroid cancer cells are more aggressive and metastasize fast, which is an important factor affecting the prognosis [2].

The pathogenesis of tumors is a multifactor and multistep process [3]. Trefoil factor 3 (TFF3) is a member of the trefoil peptide family. It was first discovered in the rat jejunum by Suemori et al. in 1991 [4]. Its genes, TFFI and TFF2, are densely packed on chromosome 21q22.3. Under physiological conditions, TFF3 is mainly distributed in the goblet cells of the small intestine and colon and plays an important role in the protection of the gastrointestinal mucosa and the repair of ulcers [4]. In recent years, studies have shown that its abnormal expression in breast, stomach, and colon tumors may be related to the occurrence and development of tumors [5–7]. Its high expression is believed to be closely related to tumor cell regional metastasis. Interleukin 6 (IL-6) is an inflammatory factor with multiple functions [8]. It can not only regulate immune and inflammatory response but also has been proven to promote the motility of liver cancer, esophageal cancer, colon cancer, renal cell carcinoma, pancreatic cancer, and oral squamous cell carcinoma. STAT3 exists in the cytoplasm before activation and is tightly regulated. Under normal circumstances, its activation is a rapid and short-term process. Under the action of cytokines and carcinogenic factors, it continues to activate to form pSTAT3 and then translocates to the nucleus. Therefore, STAT3 is overexpressed in the nucleus of tumor cells, which promotes cell proliferation and malignant transformation, hinders cell apoptosis, and exhibits carcinogenic effects [9]. Recent molecular studies have also shown that STAT3 is involved in the occurrence of a variety of tumors such as head and neck cancer, prostate cancer, breast cancer, EBV-related tumors, and various leukemias [10–14].

We are now focusing on using the understanding of TFF3 function in the development of thyroid cancer to focus on providing new ideas and prospects for early diagnosis and clinical prevention of thyroid cancer.

## 2. Materials and Methods

### 2.1. Bioinformatics

The differentially expressed TFF3 in the thyroid cancer tissues and normal tissues was analyzed by UALCAN (http://ualcan.path.uab.edu/) [15]. UALCAN was also applied for analyzing the expression of TFF3 in the patients with different cancer stages and metastasis status. In addition, the Kaplan–Meier overall survival curve for patients with thyroid cancer classified according to relative TFF3 expression was analyzed by the Gene Expression Profiling Interactive Analysis (GEPIA) website (http://gepia2.cancer-pku.cn) [16]. The database used by UALCAN and GEPIA was obtained from The Cancer Genome Atlas (TCGA, https://cancergenome.nih.gov/) databases. To explore the underlying mechanism through which TFF3 modulates the progression of thyroid cancer, Gene Set Enrichment Analysis (GSEA) [17] was performed based on TCGA thyroid cancer datasets and conducted using the Hallmark gene set dataset.

### 2.2. RT-PCR

The total RNA of cells was extracted by the TRIzol method, and RT-PCR was performed after reverse transcription into cDNA. The ABI 7500 real-time PCR system was used for detection with GAPDH mRNA as the internal reference gene. The total reaction system was 20 *μ*l: 2x UltraSYBR mixture, 10 *μ*l; the upstream and downstream primers, 0.6 *μ*l; and the cDNA template, 1 *μ*l. The reaction conditions were as follows: predenaturation at 95°C for 10 min, 40 amplification cycles (95°C, 15 s; 60°C, 1 min), and fluorescence signal collected at 60°C. The relative level of each factor mRNA was calculated via the 2^−ΔΔct^ method. The internal reference gene and target gene PCR primers, TFF3-F 5′-CCCTGCAGGAAGCAGAATGC-3′, TFF3-R 5′-CGAAGAACTGTCCTCGGGTG-3′, GAPDH-F 5′-GTTGCAACCGGGAAGGAAAT-3′, and GAPDH-R 5′-GCCCAATACGACCAAATCAGA-3′, were used.

### 2.3. Cells and Transfection

The cell lines Nthy-ori 3-1, FTC133, BCPAP, TPC-1, 8505C, and SW579 were placed in DMEM medium containing 10% FBS. Then, 2 × 10^6^ cells/dish were inoculated in a 60 mm diameter Petri dish. After 24–36 hours, the cells grew to a confluence rate of 70%. Gene transfection was carried out according to the protocols of Lipofectamine 3000 (Invitrogen, USA). The experiment was sorted into 5 groups: control group, pcDNA-NC group (cells transfected with empty vector), pcDNA-TFF3 group (cells transfected with TFF3 overexpression vector), si-NC group (cells transfected with siRNA), and si-TFF3 group (cells transfected with siRNA-TFF3). The pcDNA-TFF3, siRNA-TFF3, and their negative controls were supplied by GenePharma, Shanghai, China.

### 2.4. Colony Formation Assay

Cells were implanted in a 6 cm culture dish at 2 × 10^3^ cells/well. After 14 days, colonies were formed, washed with PBS and fixed with methanol for 15 min, and washed with PBS and crystallized violet staining for 30 min. Each experiment had 3 duplicates.

### 2.5. EdU Incorporation Assay

Cells in the control group, pcDNA-NC group, pcDNA-TFF3 group, si-NC group, and si-TFF3 group were spread on a 96-well plate. After the cells adhered, the cells were cultured for 48 h. Following the operating steps of the EdU detection kit, EdU reagent was added, incubated for 2 h, and nucleus of cells were stained with the DAPI reagent for 5 min. Under a fluorescence microscope, 5 cell images were taken and counted by the random field method. This experiment was performed in triplicate.

### 2.6. Cell Apoptosis Assay

The apoptosis of cells was determined using Annexin-V-FITC Detection Kit (BioVision, USA). Cells were (2 × 10^5^ in each well) collected and were resuspended in binding buffer. Then, Annexin-V-FITC (5 *μ*l) was added to the cells and incubated in the dark for 30 min at 4°C. Subsequently, the cells were stained with propidium iodide (PI, 5 *μ*l) for 5 min at room temperature. Finally, cell apoptosis was analyzed by using a FACScan flow cytometer (BD Biosciences, USA). Cell apoptosis assay was repeated 3 times.

### 2.7. Western Blot Assay

Cellular protein was extracted, and the protein sample with the same content and 5× loading buffer were mixed in a 4 : 1 volume and boiled in water for 5 min. After loading SDS-PAGE electrophoresis, the samples were electrotransferred to the PVDF membrane, incubated with 5% nonfat milk. Then, anti-TFF3 (cat#ab108599, 1 : 500, Abcam, UK), anti-Bcl-2 (cat#12789-1-AP, 1 : 1000, ProteinTech, USA), anti-Bax (cat#50599-2-Ig, 1 : 1000, ProteinTech, USA), anti-cleaved-caspase-3 (cat# 19677-1-AP, 1 : 500, ProteinTech, USA), anti-vimentin (cat#10366-1-AP, 1 : 1000, ProteinTech, USA), anti-N-cadherin (cat#22018-1-AP, 1 : 1000, ProteinTech, USA), anti-E-cadherin (cat#20874-1-AP, 1 : 500, ProteinTech, USA), anti-IL-6 (cat#66146-1-Ig, 1 : 1000, ProteinTech, USA), anti-JAK2 (cat#17670-1-AP, 1 : 500, ProteinTech, USA), anti-p-JAK2 (cat#8224S, 1 : 300, Cell Signaling, USA), anti-STAT3 (cat#10253-2-AP, 1 : 500, ProteinTech, USA), anti-pSTAT3 (cat#9131L, 1 : 300, Cell Signaling, USA), and anti-GAPDH (cat#10494-1-AP, 1 : 1000, ProteinTech, USA) were used as primary antibodies. After washing the membrane, 1 : 5,000 diluted secondary antibody was added for 1 h on a shaker at room temperature. After washing the membrane, ECL (Solarbio Science & Technology, Beijing, China) was used to enhance luminescence. The experiment was repeated 3 times independently.

### 2.8. Transwell Assay

The transwell chamber was placed in the wells of a 24-well culture plate. Matrigel was added to serum-free DMEM culture medium in a precooled centrifuge tube; the mixture is spread on the side of the chamber and placed in a 37°C incubator for air-drying for 1 h. 2 × 10^5^ cells were added to 200 *μ*l of serum-free DMEM to culture in the upper chamber. After 24 h of incubation, the cell supernatant was aspirated and the cells on the upper chamber side of the microporous membrane were gently wiped. After washing with PBS and staining with crystal violet, 5 fields of view were counted and averaged. For the migration assay, the procedure was similar to the invasion experiment, except that the uncoated filters were applied in the transwell chamber. This experiment was performed in triplicate.

### 2.9. Statistical Analysis

Experimental data were expressed as mean ± standard deviation and analyzed statistically via SPSS 19.0. Analysis of variance was used for the values between groups. *P* < 0.05 was regarded as statistically significant.

## 3. Results

### 3.1. TFF3 Is Underexpressed in Thyroid Cancer Cell Lines and Tumors

**Figure 1:**
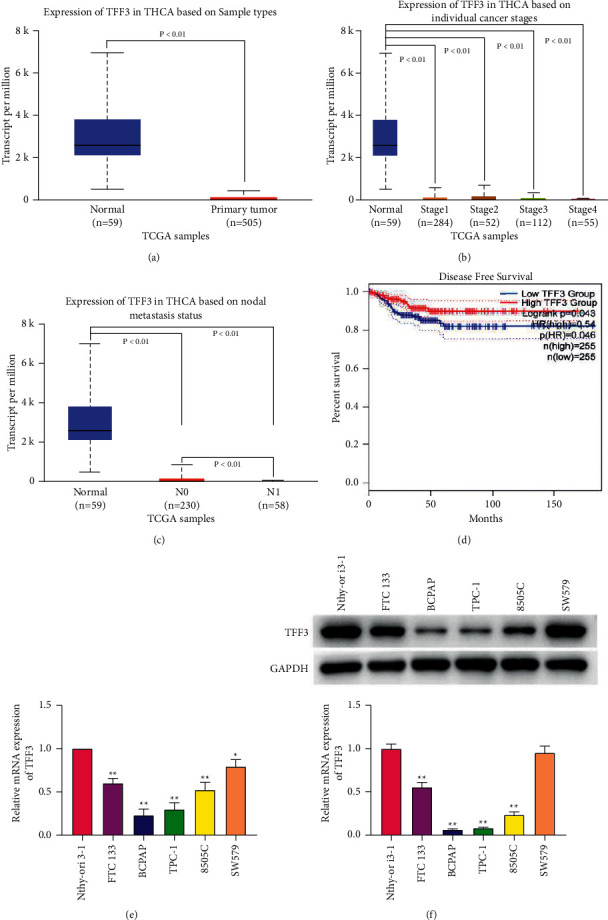
TFF3 is underexpressed in thyroid cancer cell lines and tumors. (a) The expression of TFF3 in thyroid cancer tissues (*n* = 505) and the normal tissues (*n* = 59) analyzed by UALCAN based on the TCGA database. (b) The expression of TFF3 in normal tissues (*n* = 59) and thyroid cancer stage 1 (*n* = 284), stage 2 (*n* = 52), stage 3 (*n* = 112), and stage 4 tissues (*n* = 55) analyzed by UALCAN based on the TCGA database. (c) TFF3 expression level in thyroid cancer nodal metastasis *N*0 and *N*1 tissues and the normal tissues analyzed by UALCAN based on the TCGA database. (d) GEPIA analysis of disease-free survival of TFF3. (e) RT-PCR analysis of the expression level of TFF3 in 5 cancerous cell lines, FTC133, BCPAP, TPC-1, 8505C, and SW579, and a normal thyroid cell line Nthy-ori 3-1. (f) Western blot analysis of the expression level of TFF3 in 5 cancerous cell lines, FTC133, BCPAP, TPC-1, 8505C, and SW579, and a normal thyroid cell line Nthy-ori 3-1.^*∗*^*P* < 0.05 and ^∗∗^*P* < 0.01 vs. Nthy-ori 3-1.

First, the expression and prognosis value of TFF3 were analyzed by UALCAN and GEPIA websites based on the TCGA database. The results of Figure 1(a) show that TFF3 expression was significantly downregulated in thyroid cancer tissues in comparison to the normal tissues. Then, we observed high level of TFF3 in normal tissues and low expression of TFF3 in the tissues of thyroid cancer stage 1, stage 2, stage 3, and stage 4 (*P* < 0.01; Figure 1(b)). TFF3 level in thyroid cancer nodal metastasis tissues was prominently downregulated compared with that in the normal tissues, and the level of TFF3 in thyroid cancer nodal metastasis *N*0 was overexpressed compared with that in nodal metastasis *N*1 (*P* < 0.01; Figure 1(c)). Moreover, the results of Figure 1(d) show that patients with low TFF3 expression showed worse disease-free survival than those with higher TFF3 expression (*P*=0.046 < 0.05).

Next, in order to evaluate the level of TFF3 in cancerous and normal thyroid cells, its mRNA and protein levels were tested in 5 cancerous cell lines, FTC133, BCPAP, TPC-1, 8505C, and SW579, and a normal thyroid cell line Nthy-ori 3-1 using RT-PCR and western blot. As shown in Figure 1(e), the mRNA levels of TFF3 in 5 cancerous cell lines were markedly reduced (*P* < 0.05 and *P* < 0.01). Moreover, the protein expression of TFF3 was decreased only in FTC133, BCPAP, TPC-1, and 8505C cells (Figure 1(f); *P* < 0.01). BCPAP and TPC-1 cell lines with the lowest mRNA and protein levels were used in follow-up experiments (*P* < 0.01).

### 3.2. Underexpressed TFF3 Increases Cell Proliferation

**Figure 2:**
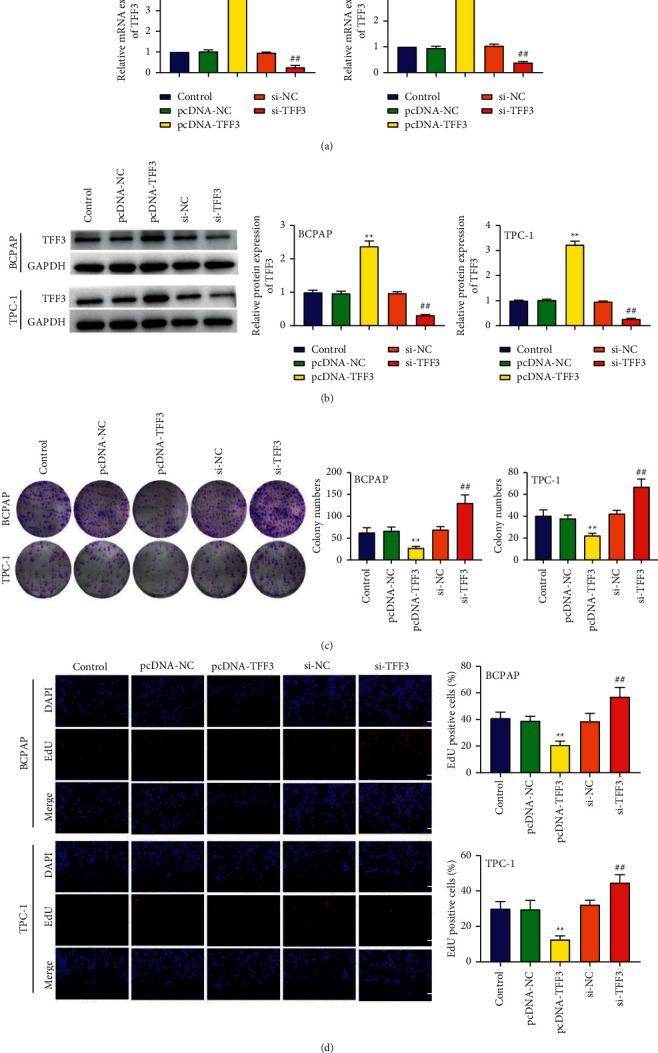
Underexpressed TFF3 increases cell proliferation. (a) RT-PCR analysis of the expression of TFF3 of BCPAP and TPC-1 cells in the pcDNA-TFF3 group, the control group, the pcDNA-NC group, the si-TFF3 group, and the si-NC group. (b) Western blot analysis of the expression of TFF3 of BCPAP and TPC-1 cells in the pcDNA-TFF3 group, the control group, the pcDNA-NC group, the si-TFF3 group, and the si-NC group. (c) Colony formation analysis of colony ability of BCPAP and TPC-1 cells. (d) EdU incorporation analysis of the proliferation ability of BCPAP and TPC-1 cells. ^∗∗^*P* < 0.01 vs. control and pcDNA-NC; ^##^*P* < 0.01 vs. control and si-NC.

As shown in Figures 2(a) and 2(b), the transfection efficiency of pcDNA-TFF3 and si-TFF3 in BCPAP and TPC-1 cells was confirmed by RT-PCR and western blot. The colony formation and EdU incorporation assays were available for determining the role of TFF3 in cell proliferation. We found that decreased expression of TFF3 significantly increased the colony numbers of BCPAP and TPC-1 cells, while increased expression of TFF3 markedly decreased the cells' colony numbers (*P* < 0.01; Figure 2(c)). Subsequently, EdU incorporation assay also showed similar results that comparing with the si-NC group, the cell proliferation of the si-TFF3 group was strongly increased (*P* < 0.01); on the contrary, the cell proliferation of the pcDNA-TFF3 group was significantly decreased compared to the pcDNA-NC group (*P* < 0.01; Figure 2(d)).

### 3.3. Underexpressed TFF3 Inhibits Cell Apoptosis

**Figure 3:**
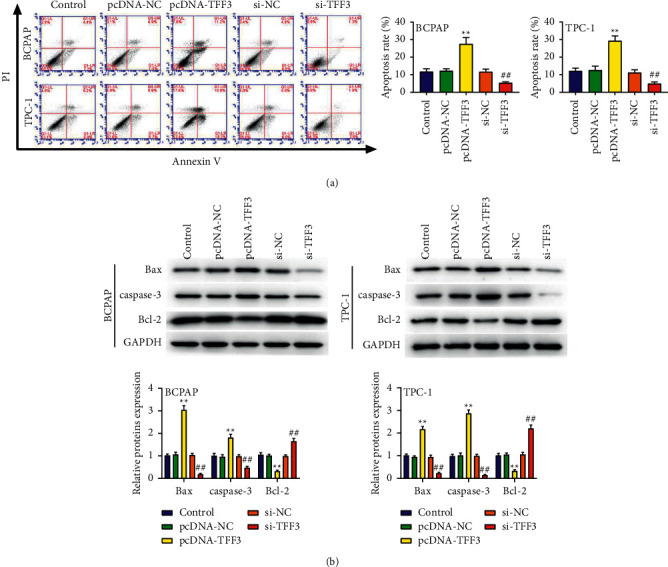
Underexpressed TFF3 inhibits cell apoptosis. (a) Flow cytometry analysis of the rate of BCPAP and TPC-1 cell apoptosis. (b) Western blot analysis of the protein of Bax and caspase-3 and Bcl-2. ^∗∗^*P* < 0.01 vs. control and pcDNA-NC; ^##^*P* < 0.01 vs. control and si-NC.

To assess the effect of TFF3 on the apoptosis ability, we performed flow cytometry and western blot assays. The results in Figure 3(a) show that the rate of apoptosis in the si-TFF3 group was strongly decreased compared to the si-NC group and that in the pcDNA-TFF3 group was markedly increased compared to the pcDNA-NC group (*P* < 0.01). The western blot results in Figure 3(b) show that cells had higher protein of Bax and caspase-3 and Bcl-2 was underexpressed in the pcDNA-TFF3 group in comparison to the pcDNA-NC group. The protein levels of Bax, caspase-3, and Bcl-2 of the si-TFF3 group and pcDNA-NC group were exactly the opposite (*P* < 0.01).

### 3.4. Underexpressed TFF3 Increases Cell Migration and Invasion

**Figure 4:**
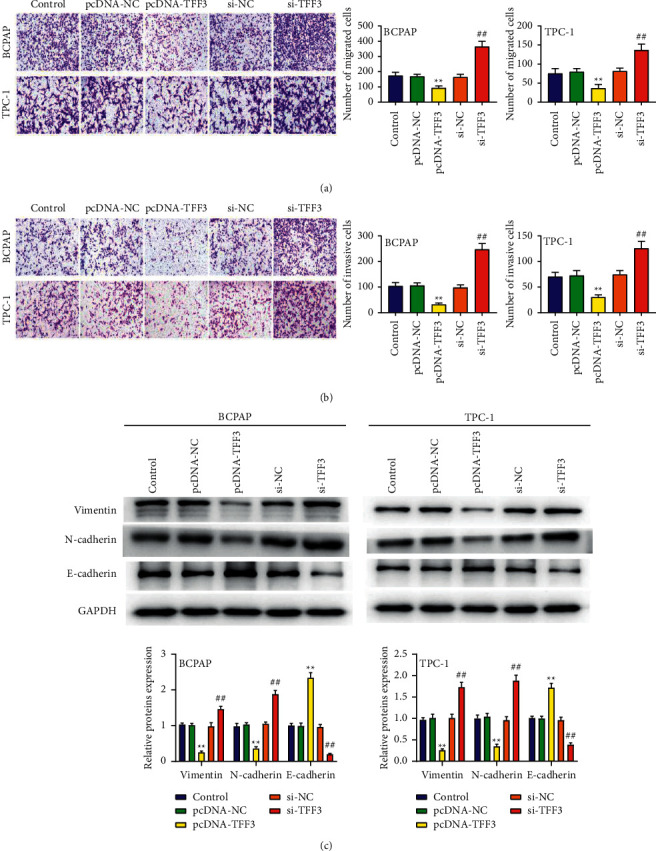
Underexpressed TFF3 increases cell migration and invasion. (a) Transwell analysis of the migration of BCPAP and TPC-1 cells. (b) Transwell analysis of the invasion of BCPAP and TPC-1 cells. (c) The protein of E-cadherin, vimentin, and N-cadherin. ^∗∗^*P* < 0.01 vs. control and pcDNA-NC; ^##^*P* < 0.01 vs. control and si-NC.

We further analyzed the effect of TFF3 on cell migration and invasion ability. The results in Figures 4(a) and 4(b) show that the migration and invasion of BCPAP and TPC-1 cells were prominently inhibited in the pcDNA-TFF3 group and markedly increased in the si-TFF3 group (*P* < 0.01). Simultaneously, the level of E-cadherin was induced while vimentin and N-cadherin were downregulated in the pcDNA-TFF3 group in comparison to the pcDNA-NC group, and the expression of E-cadherin was prominently decreased while vimentin and N-cadherin were upregulated in the si-TFF3 group in comparison to the si-NC group in BCPAP and TPC-1 cells (*P* < 0.01; Figure 4(c)).

### 3.5. TFF3 Inhibits Thyroid Cancer Cell Progression Related to IL-6/JAK/STAT3 Signaling Pathway

**Figure 5:**
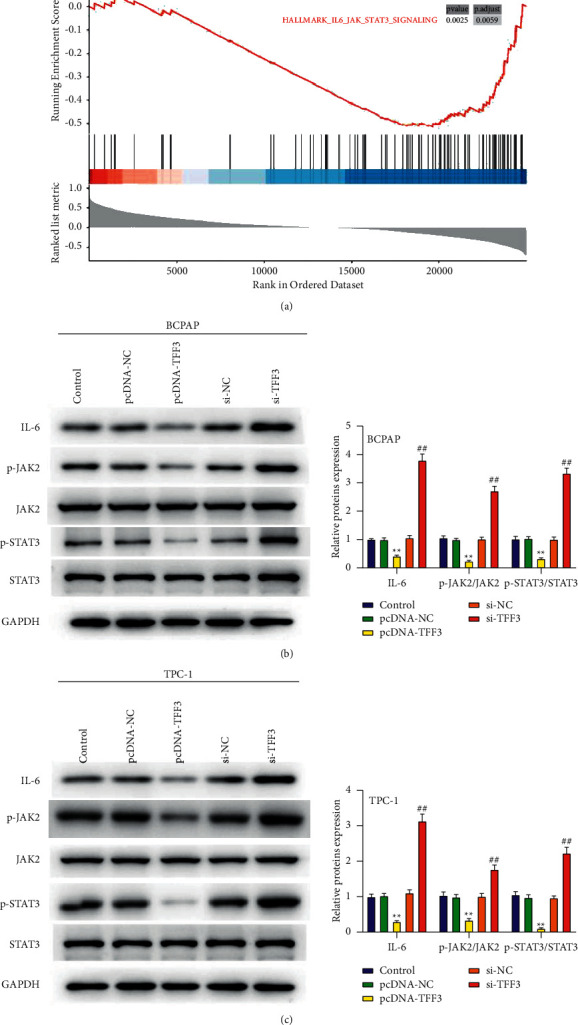
TFF3 inhibits thyroid cancer cell progression related to the IL-6/JAK/STAT3 signaling pathway. (a) GSEA pathway analysis of the mechanism by which TFF3 inhibits the progression of thyroid cancer cells. (b, c) The protein of IL-6, p-JAK2, JAK2, pSTAT3, and STAT3 in BCPAP and TPC-1 cells. ^∗∗^*P* < 0.01 vs. control and pcDNA-NC; ^##^*P* < 0.01 vs. control and si-NC.

To clarify the mechanism by which TFF3 inhibits the progression of thyroid cancer cells, we performed GSEA pathway analysis. The results revealed in Figure 5(a) show that TFF3 inhibited the IL-6/JAK/STAT3 signaling pathway. We further analyzed the effect of TFF3 on the IL-6/JAK/STAT3 signaling pathway using western blot assay. The results revealed in Figures 5(b) and 5(c) show that the levels of IL-6, p-JAK2/JAK2, and pSTAT3/STAT3 were induced in the pcDNA-TFF3 group compared to the pcDNA-NC group, while there is upregulation of IL-6, p-JAK2/JAK2, and pSTAT3/STAT3 in the si-TFF3 group in comparison to the si-NC group in BCPAP and TPC-1 cells (*P* < 0.01). The IL-6/JAK/STAT3 signaling pathway might be involved in the effect of TFF3 on thyroid cancer cell progression.

## 4. Discussion

At present, the evaluation of the prognosis of patients with thyroid cancer mainly distinguishes the high-risk group and the low-risk group based on the age of the patient, the size of the tumor, and whether it has metastasis to a distant location and lacks some clear biological indicators. Therefore, understanding the molecular mechanism of thyroid cancer metastasis and designing specific treatment and prognostic evaluation programs for each link of tumor invasion and metastasis, so as to improve the clinical treatment effect and patient survival rate, have become a hot issue in the current research field of thyroid cancer [18].

The role of TFF3 in tumors has received a lot of attention lately. TFF3 plays a role in tumors by inhibiting cell adhesion, promoting cell invasion, inhibiting apoptosis, and promoting neovascularization. Studies have shown that TFF3, as an important cytokine in the gastrointestinal mucosa, is involved in the process of gastric mucosal adenoma formation, intestinal metaplasia, dysplasia, and malignant transformation [7, 19, 20]. It may be an early molecular event in the process of gastric mucosal carcinogenesis and can affect gastric cancer angiogenesis and promote tumor invasion and metastasis [21]. The results of this study indicated that TFF3 inhibited the motility of thyroid cancer cells and promoted cell apoptosis. However, the binding protein of TFF3 has not yet been clarified, and its intracellular mechanism of action and related research on specific signal transduction pathways are still in the initial stage. With the continuous development of molecular biology, we can further understand the relationship between TFF3 and the occurrence and development of the thyroid, which is conducive to the early diagnosis and treatment of thyroid cancer.

After IL-6 binds to its receptor, it can exert biological functions through three signal pathways: JAK/STAT pathway, Ras/ERK pathway, and PI3K-mediated pathway [22, 23]. Among them, it has been confirmed in many human tumors that, after IL-6 binds to its receptor (two subunits IL-6R*α* and gp130), gp130 activates STAT3 transcription factor through phosphorylation of JAK, and phosphorylated STAT3 forms dimers and metastasizes [24]. In the nucleus, the JAK/STAT3 signaling pathway is continuously activated to cause uncontrolled tumor cell proliferation, resist apoptosis, support angiogenesis, and evade immune surveillance [25, 26]. This study found that downregulated expression of TFF3 increases cell proliferation, migration, and invasion and upregulation of IL-6, p-JAK2/JAK2, and pSTAT3/STAT3 in the si-TFF3 group in BCPAP and TPC-1 cells.

Studies have shown that the expression level of STAT3 in normal thyroid tissue, thyroid adenoma, and thyroid cancer sequentially increases, suggesting that the increase in STAT3 expression level is closely related to the occurrence of thyroid cancer [27]. The study also found that the expression level of STAT3 in cases with lymph node metastasis was uncommonly higher than that in the group without lymph node metastasis, and the higher the stage of thyroid cancer, the higher the positive rate of STAT3 [28, 29]. In this study, the expression pattern of TFF3 was opposite to that of STAT3. The expression level of TFF3 in thyroid cancer was lower than that in normal thyroid tissue. It was also found that the expression level of TFF3 in cases with lymph node metastasis was significantly lower than that in the non-lymph node metastasis group. Downregulated expression of TFF3 increases the expression of pSTAT3/STAT3, suggesting that the effect of TFF3 expression level on the occurrence of thyroid cancer is closely related to the expression of STAT3.

In summary, the TFF3 detection of thyroid cancer is helpful to evaluate the invasion and metastasis of thyroid cancer and provides a reference for the clinical treatment and prognosis of thyroid cancer.

## Data Availability

The data used to support the findings of this study are available from the corresponding author upon reasonable request.

## Supplementary Materials

Supplementary Materials The original experimental data of the manuscript are included in the supplementary files named “original data” and “original files.”
