# Supplementary material for: Trefoil Factor 3 Inhibits Thyroid Cancer Cell Progression Related to IL-6/JAK/STAT3 Signaling Pathway
Source: Evid Based Complement Alternat Med. 2021 Sep 14;2021:2130229. doi: 10.1155/2021/2130229 (PMC8457945; doi:10.1155/2021/2130229)
Supplement: Supplementary Materials — The original experimental data of the manuscript are included in the supplementary files named “original data” and “original files.” [file 2130229.f1.zip › 2130229.f1/original data.docx]

**1E**

| PCR |  |  | Nthy-ori 3-1 | FTC 133 | BCPAP | TPC-1 | 8505C | SW579 |
| --- | --- | --- | --- | --- | --- | --- | --- | --- |
|  |  |  | 1 | 0.54 | 0.21 | 0.21 | 0.5 | 0.8 |
|  |  |  | 1 | 0.65 | 0.31 | 0.32 | 0.62 | 0.71 |
|  |  |  | 1 | 0.61 | 0.17 | 0.36 | 0.45 | 0.87 |
|  |  |  |  |  |  |  |  |  |
|  |  |  |  |  |  |  |  |  |
| **第一次** | **GAPDH** | **TFF3** |  | **Average** | **Average** | **△CT** | **△△CT** | **2-△△CT** |
| Nthy-ori 3-1 | 23.46 | 25.85 |  |  |  |  |  |  |
|  | 23.28 | 25.06 |  |  |  |  |  |  |
|  | 23.11 | 25.20 |  | 23.28 | 25.37 | 2.09 | 0.00 | 1.00 |
| FTC 133 | 23.02 | 26.45 |  |  |  |  |  |  |
|  | 22.89 | 25.75 |  |  |  |  |  |  |
|  | 23.31 | 25.92 |  | 23.07 | 26.04 | 2.97 | 0.88 | 0.54 |
| BCPAP | 24.15 | 28.95 |  |  |  |  |  |  |
|  | 24.39 | 28.87 |  |  |  |  |  |  |
|  | 24.32 | 27.98 |  | 24.29 | 28.60 | 4.31 | 2.22 | 0.21 |
| TPC-1 | 23.74 | 27.75 |  |  |  |  |  |  |
|  | 23.67 | 27.91 |  |  |  |  |  |  |
|  | 23.65 | 28.44 |  | 23.69 | 28.03 | 4.34 | 2.25 | 0.21 |
| 8505C | 24.29 | 27.16 |  |  |  |  |  |  |
|  | 24.01 | 26.91 |  |  |  |  |  |  |
|  | 24.34 | 27.84 |  | 24.21 | 27.30 | 3.09 | 1.00 | 0.50 |
| SW579 | 22.54 | 24.39 |  |  |  |  |  |  |
|  | 22.38 | 24.75 |  |  |  |  |  |  |
|  | 22.89 | 25.93 |  | 22.60 | 25.02 | 2.42 | 0.33 | 0.80 |
|  |  |  |  |  |  |  |  |  |
| **第二次** | **GAPDH** | **TFF3** |  | **Average** | **Average** | **△CT** | **△△CT** | **2-△△CT** |
| Nthy-ori 3-1 | 18.69 | 20.40 |  |  |  |  |  |  |
|  | 17.46 | 20.60 |  |  |  |  |  |  |
|  | 17.63 | 20.25 |  | 17.93 | 20.42 | 2.49 | 0.00 | 1.00 |
| FTC 133 | 16.48 | 19.91 |  |  |  |  |  |  |
|  | 16.63 | 20.11 |  |  |  |  |  |  |
|  | 17.38 | 19.78 |  | 16.83 | 19.93 | 3.10 | 0.61 | 0.65 |
| BCPAP | 17.12 | 21.52 |  |  |  |  |  |  |
|  | 17.24 | 21.81 |  |  |  |  |  |  |
|  | 18.03 | 21.56 |  | 17.46 | 21.63 | 4.17 | 1.68 | 0.31 |
| TPC-1 | 17.29 | 22.15 |  |  |  |  |  |  |
|  | 18.12 | 22.03 |  |  |  |  |  |  |
|  | 18.22 | 21.89 |  | 17.88 | 22.02 | 4.15 | 1.66 | 0.32 |
| 8505C | 19.53 | 21.94 |  |  |  |  |  |  |
|  | 19.06 | 22.80 |  |  |  |  |  |  |
|  | 19.25 | 22.65 |  | 19.28 | 22.46 | 3.18 | 0.69 | 0.62 |
| SW579 | 18.75 | 20.91 |  |  |  |  |  |  |
|  | 18.62 | 21.64 |  |  |  |  |  |  |
|  | 18.19 | 21.98 |  | 18.52 | 21.51 | 2.99 | 0.50 | 0.71 |
|  |  |  |  |  |  |  |  |  |
| **第三次** | **GAPDH** | **TFF3** |  | **Average** | **Average** | **△CT** | **△△CT** | **2-△△CT** |
| Nthy-ori 3-1 | 19.63 | 22.80 |  |  |  |  |  |  |
|  | 19.76 | 22.47 |  |  |  |  |  |  |
|  | 20.21 | 22.86 |  | 19.87 | 22.71 | 2.84 | 0.00 | 1.00 |
| FTC 133 | 20.17 | 24.23 |  |  |  |  |  |  |
|  | 20.76 | 24.25 |  |  |  |  |  |  |
|  | 20.74 | 23.84 |  | 20.56 | 24.11 | 3.55 | 0.71 | 0.61 |
| BCPAP | 18.53 | 23.31 |  |  |  |  |  |  |
|  | 17.07 | 23.74 |  |  |  |  |  |  |
|  | 18.23 | 22.86 |  | 17.94 | 23.30 | 5.36 | 2.52 | 0.17 |
| TPC-1 | 17.26 | 21.64 |  |  |  |  |  |  |
|  | 17.32 | 21.71 |  |  |  |  |  |  |
|  | 17.39 | 21.57 |  | 17.32 | 21.64 | 4.32 | 1.48 | 0.36 |
| 8505C | 19.05 | 23.69 |  |  |  |  |  |  |
|  | 19.74 | 23.19 |  |  |  |  |  |  |
|  | 19.31 | 23.16 |  | 19.37 | 23.35 | 3.98 | 1.14 | 0.45 |
| SW579 | 16.95 | 19.05 |  |  |  |  |  |  |
|  | 17.14 | 20.19 |  |  |  |  |  |  |
|  | 16.88 | 20.86 |  | 16.99 | 20.03 | 3.04 | 0.20 | 0.87 |

**1F**

| western blot |  |  | Nthy-ori 3-1 | FTC 133 | BCPAP | TPC-1 | 8505C | SW579 |
| --- | --- | --- | --- | --- | --- | --- | --- | --- |
|  |  |  | 1 | 0.55 | 0.06 | 0.08 | 0.23 | 1.04 |
|  |  |  | 1.05 | 0.61 | 0.07 | 0.09 | 0.27 | 0.9 |
|  |  |  | 0.94 | 0.5 | 0.05 | 0.07 | 0.2 | 0.91 |
|  |  |  |  |  |  |  |  |  |

**2A**

| PCR |  | BCPAP |  |  |  |  |  |  |  | TPC-1 |  |  |  |  |  |  |  |
| --- | --- | --- | --- | --- | --- | --- | --- | --- | --- | --- | --- | --- | --- | --- | --- | --- | --- |
|  |  |  | Control | pcDNA-NC | pcDNA-TFF3 | si-NC | si-TFF3 |  |  |  | Control | pcDNA-NC | pcDNA-TFF3 | si-NC | si-TFF3 |  |  |
|  |  |  | 1 | 0.95 | 3.65 | 0.95 | 0.24 |  |  |  | 1 | 0.9 | 3.24 | 1.11 | 0.35 |  |  |
|  |  |  | 1 | 1.05 | 4.23 | 0.93 | 0.35 |  |  |  | 1 | 0.92 | 2.58 | 0.99 | 0.43 |  |  |
|  |  |  | 1 | 1.09 | 3.86 | 1 | 0.18 |  |  |  | 1 | 1.03 | 2.96 | 1.04 | 0.39 |  |  |
|  |  |  |  |  |  |  |  |  |  |  |  |  |  |  |  |  |  |
|  |  |  |  |  |  |  |  |  |  |  |  |  |  |  |  |  |  |
|  |  |  |  |  |  |  |  |  |  |  |  |  |  |  |  |  |  |
| **第一次** | **组别** | **GAPDH** | **TFF3** | **Average** | **Average** | **△CT** | **△△CT** | **2-△△CT** | | **组别** | **GAPDH** | **TFF3** | **Average** | **Average** | **△CT** | **△△CT** | **2-△△CT** |
|  | Control | 16.69 | 18.76 |  |  |  |  |  |  | Control | 21.24 | 23.79 |  |  |  |  |  |
|  |  | 17.67 | 19.77 |  |  |  |  |  |  |  | 20.31 | 22.16 |  |  |  |  |  |
|  |  | 18.84 | 19.99 | 17.73 | 19.51 | 1.77 | 0.00 | 1.00 |  |  | 20.12 | 23.23 | 20.56 | 23.06 | 2.50 | 0.00 | 1.00 |
|  | pcDNA-NC | 19.03 | 21.23 |  |  |  |  |  |  | pcDNA-NC | 19.52 | 21.25 |  |  |  |  |  |
|  |  | 18.32 | 20.02 |  |  |  |  |  |  |  | 19.24 | 22.52 |  |  |  |  |  |
|  |  | 18.68 | 20.31 | 18.68 | 20.52 | 1.84 | 0.07 | 0.95 |  |  | 19.11 | 22.05 | 19.29 | 21.94 | 2.65 | 0.15 | 0.90 |
|  | pcDNA-TFF3 | 17.18 | 17.15 |  |  |  |  |  |  | pcDNA-TFF3 | 18.30 | 18.74 |  |  |  |  |  |
|  |  | 18.29 | 17.27 |  |  |  |  |  |  |  | 18.19 | 18.96 |  |  |  |  |  |
|  |  | 17.11 | 17.87 | 17.53 | 17.43 | -0.10 | -1.87 | 3.65 |  |  | 18.31 | 19.51 | 18.27 | 19.07 | 0.80 | -1.70 | 3.24 |
|  | si-NC | 18.77 | 20.15 |  |  |  |  |  |  | si-NC | 20.31 | 21.78 |  |  |  |  |  |
|  |  | 18.91 | 20.71 |  |  |  |  |  |  |  | 19.28 | 21.28 |  |  |  |  |  |
|  |  | 17.87 | 20.22 | 18.52 | 20.36 | 1.84 | 0.07 | 0.95 |  |  | 19.07 | 22.64 | 19.55 | 21.90 | 2.35 | -0.15 | 1.11 |
|  | si-TFF3 | 17.8 | 21.72 |  |  |  |  |  |  | si-TFF3 | 18.53 | 22.39 |  |  |  |  |  |
|  |  | 16.34 | 21.71 |  |  |  |  |  |  |  | 18.37 | 22.96 |  |  |  |  |  |
|  |  | 18.63 | 20.84 | 17.59 | 21.42 | 3.83 | 2.06 | 0.24 |  |  | 18.31 | 21.86 | 18.40 | 22.40 | 4.00 | 1.50 | 0.35 |
|  |  |  |  |  |  |  |  |  |  |  |  |  |  |  |  |  |  |
|  |  |  |  |  |  |  |  |  |  |  |  |  |  |  |  |  |  |
|  |  |  |  |  |  |  |  |  |  |  |  |  |  |  |  |  |  |
| **第二次** | **组别** | **GAPDH** | **TFF3** | **Average** | **Average** | **△CT** | **△△CT** | **2-△△CT** | | **组别** | **GAPDH** | **TFF3** | **Average** | **Average** | **△CT** | **△△CT** | **2-△△CT** |
|  | Control | 19.52 | 21.16 |  |  |  |  |  |  | Control | 19.73 | 22.32 |  |  |  |  |  |
|  |  | 18.6 | 20.14 |  |  |  |  |  |  |  | 19.12 | 22.55 |  |  |  |  |  |
|  |  | 18.33 | 20.87 | 18.82 | 20.72 | 1.91 | 0.00 | 1.00 |  |  | 19.37 | 22.01 | 19.41 | 22.29 | 2.89 | 0.00 | 1.00 |
|  | pcDNA-NC | 16.77 | 18.72 |  |  |  |  |  |  | pcDNA-NC | 16.32 | 19.56 |  |  |  |  |  |
|  |  | 16.72 | 18.66 |  |  |  |  |  |  |  | 16.56 | 20.09 |  |  |  |  |  |
|  |  | 17.21 | 18.82 | 16.90 | 18.73 | 1.83 | -0.08 | 1.05 |  |  | 17.14 | 19.4 | 16.67 | 19.68 | 3.01 | 0.12 | 0.92 |
|  | pcDNA-TFF3 | 17.08 | 17.56 |  |  |  |  |  |  | pcDNA-TFF3 | 18.07 | 19.07 |  |  |  |  |  |
|  |  | 17.21 | 16.73 |  |  |  |  |  |  |  | 17.53 | 19.01 |  |  |  |  |  |
|  |  | 17.1 | 16.59 | 17.13 | 16.96 | -0.17 | -2.08 | 4.23 |  |  | 17.2 | 19.29 | 17.60 | 19.12 | 1.52 | -1.37 | 2.58 |
|  | si-NC | 17.54 | 19.86 |  |  |  |  |  |  | si-NC | 17.2 | 19.54 |  |  |  |  |  |
|  |  | 18.02 | 19.78 |  |  |  |  |  |  |  | 17.25 | 20.39 |  |  |  |  |  |
|  |  | 17.89 | 19.86 | 17.82 | 19.83 | 2.02 | 0.11 | 0.93 |  |  | 17.14 | 20.36 | 17.20 | 20.10 | 2.90 | 0.01 | 0.99 |
|  | si-TFF3 | 18.37 | 21.59 |  |  |  |  |  |  | si-TFF3 | 17.62 | 21.65 |  |  |  |  |  |
|  |  | 18.36 | 21.89 |  |  |  |  |  |  |  | 17.49 | 21.68 |  |  |  |  |  |
|  |  | 18.28 | 21.79 | 18.34 | 21.76 | 3.42 | 1.51 | 0.35 |  |  | 17.88 | 21.96 | 17.66 | 21.76 | 4.10 | 1.21 | 0.43 |
|  |  |  |  |  |  |  |  |  |  |  |  |  |  |  |  |  |  |
| **第三次** | **组别** | **GAPDH** | **TFF3** | **Average** | **Average** | **△CT** | **△△CT** | **2-△△CT** | | **组别** | **GAPDH** | **TFF3** | **Average** | **Average** | **△CT** | **△△CT** | **2-△△CT** |
|  | Control | 15.97 | 18.09 |  |  |  |  |  |  | Control | 17.02 | 21.21 |  |  |  |  |  |
|  |  | 15.03 | 17.35 |  |  |  |  |  |  |  | 17.12 | 20.92 |  |  |  |  |  |
|  |  | 15.06 | 18.53 | 15.35 | 17.99 | 2.64 | 0.00 | 1.00 |  |  | 17.32 | 20.53 | 17.15 | 20.89 | 3.73 | 0.00 | 1.00 |
|  | pcDNA-NC | 15.72 | 18.33 |  |  |  |  |  |  | pcDNA-NC | 16.15 | 19.39 |  |  |  |  |  |
|  |  | 15.18 | 18.38 |  |  |  |  |  |  |  | 16.09 | 19.27 |  |  |  |  |  |
|  |  | 16.1 | 17.84 | 15.67 | 18.18 | 2.52 | -0.12 | 1.09 |  |  | 15.62 | 20.28 | 15.95 | 19.65 | 3.69 | -0.04 | 1.03 |
|  | pcDNA-TFF3 | 15.5 | 16.2 |  |  |  |  |  |  | pcDNA-TFF3 | 17.04 | 19.10 |  |  |  |  |  |
|  |  | 15.25 | 16.07 |  |  |  |  |  |  |  | 17.08 | 19.43 |  |  |  |  |  |
|  |  | 15.54 | 16.09 | 15.43 | 16.12 | 0.69 | -1.95 | 3.86 |  |  | 17.07 | 19.16 | 17.06 | 19.23 | 2.17 | -1.56 | 2.96 |
|  | si-NC | 16.91 | 21.03 |  |  |  |  |  |  | si-NC | 18.30 | 22.71 |  |  |  |  |  |
|  |  | 19.35 | 21.01 |  |  |  |  |  |  |  | 18.36 | 21.42 |  |  |  |  |  |
|  |  | 19.65 | 20.28 | 18.13 | 20.77 | 2.64 | 0.00 | 1.00 |  |  | 18.15 | 21.72 | 18.27 | 21.95 | 3.68 | -0.05 | 1.04 |
|  | si-TFF3 | 18.61 | 24.52 |  |  |  |  |  |  | si-TFF3 | 16.05 | 21.92 |  |  |  |  |  |
|  |  | 18.7 | 23.87 |  |  |  |  |  |  |  | 16.90 | 21.04 |  |  |  |  |  |
|  |  | 17.73 | 23.32 | 18.81 | 23.90 | 5.10 | 2.46 | 0.18 |  |  | 16.36 | 21.65 | 16.44 | 21.54 | 5.10 | 1.37 | 0.39 |
|  |  |  |  |  |  |  |  |  |  |  |  |  |  |  |  |  |  |

**2B**

| western blot |  | BCPAP |  |  |  |  |  |  |  | TPC-1 |  |  |  |  |  |
| --- | --- | --- | --- | --- | --- | --- | --- | --- | --- | --- | --- | --- | --- | --- | --- |
|  |  |  | Control | pcDNA-NC | pcDNA-TFF3 | si-NC | si-TFF3 |  |  |  | Control | pcDNA-NC | pcDNA-TFF3 | si-NC | si-TFF3 |
|  |  |  | 1 | 0.97 | 2.4 | 0.97 | 0.32 |  |  |  | 1 | 1.02 | 3.2 | 0.95 | 0.27 |
|  |  |  | 0.93 | 1.03 | 2.52 | 0.93 | 0.29 |  |  |  | 1.02 | 1.06 | 3.39 | 0.93 | 0.24 |
|  |  |  | 1.06 | 0.9 | 2.2 | 1.02 | 0.33 |  |  |  | 0.98 | 0.99 | 3.11 | 0.99 | 0.29 |
|  |  |  |  |  |  |  |  |  |  |  |  |  |  |  |  |
|  |  |  |  |  |  |  |  |  |  |  |  |  |  |  |  |

**2C**

| cell colon |  | BCPAP |  |  |  |  |  |  |  | TPC-1 |  |  |  |  |  |
| --- | --- | --- | --- | --- | --- | --- | --- | --- | --- | --- | --- | --- | --- | --- | --- |
|  |  |  | Control | pcDNA-NC | pcDNA-TFF3 | si-NC | si-TFF3 |  |  |  | Control | pcDNA-NC | pcDNA-TFF3 | si-NC | si-TFF3 |
|  |  |  | 60 | 67 | 27 | 70 | 130 |  |  |  | 40 | 38 | 20 | 43 | 67 |
|  |  |  | 75 | 75 | 31 | 76 | 149 |  |  |  | 46 | 41 | 24 | 45 | 74 |
|  |  |  | 54 | 58 | 24 | 62 | 112 |  |  |  | 35 | 35 | 23 | 39 | 60 |
|  |  |  |  |  |  |  |  |  |  |  |  |  |  |  |  |

**2D**

| EdU |  | BCPAP |  |  |  |  |  |  |  | TPC-1 |  |  |  |  |  |
| --- | --- | --- | --- | --- | --- | --- | --- | --- | --- | --- | --- | --- | --- | --- | --- |
|  |  |  | Control | pcDNA-NC | pcDNA-TFF3 | si-NC | si-TFF3 |  |  |  | Control | pcDNA-NC | pcDNA-TFF3 | si-NC | si-TFF3 |
|  |  |  | 40 | 37 | 20 | 38 | 57 |  |  |  | 30 | 29 | 12 | 32 | 45 |
|  |  |  | 46 | 43 | 24 | 45 | 64 |  |  |  | 34 | 35 | 15 | 35 | 49 |
|  |  |  | 37 | 37 | 18 | 33 | 50 |  |  |  | 26 | 25 | 11 | 30 | 40 |
|  |  |  |  |  |  |  |  |  |  |  |  |  |  |  |  |

**3A**

| flow cytometry |  | BCPAP |  |  |  |  |  |  |  | TPC-1 |  |  |  |  |  |
| --- | --- | --- | --- | --- | --- | --- | --- | --- | --- | --- | --- | --- | --- | --- | --- |
|  |  |  | Control | pcDNA-NC | pcDNA-TFF3 | si-NC | si-TFF3 |  |  |  | Control | pcDNA-NC | pcDNA-TFF3 | si-NC | si-TFF3 |
|  |  |  | 11.6 | 12.5 | 27.5 | 11.7 | 5.3 |  |  |  | 12.1 | 12.6 | 29.1 | 11 | 4.9 |
|  |  |  | 13.5 | 13.2 | 31.2 | 13.2 | 6 |  |  |  | 13.8 | 14.9 | 32.2 | 13.5 | 5.9 |
|  |  |  | 10.2 | 11 | 24 | 10 | 4.8 |  |  |  | 10.5 | 10.2 | 26.5 | 8.6 | 4 |
|  |  |  |  |  |  |  |  |  |  |  |  |  |  |  |  |

**3B**

| western blot |  | BCPAP |  |  |  |  |  |  |  | TPC-1 |  |  |  |  |  |
| --- | --- | --- | --- | --- | --- | --- | --- | --- | --- | --- | --- | --- | --- | --- | --- |
|  |  | Bax | Control | pcDNA-NC | pcDNA-TFF3 | si-NC | si-TFF3 |  |  | Bax | Control | pcDNA-NC | pcDNA-TFF3 | si-NC | si-TFF3 |
|  |  |  | 1.05 | 1.02 | 2.93 | 1.1 | 0.14 |  |  |  | 0.97 | 0.95 | 2.28 | 0.96 | 0.27 |
|  |  |  | 1.06 | 1.18 | 3.26 | 1.03 | 0.17 |  |  |  | 0.97 | 0.99 | 2.18 | 0.85 | 0.19 |
|  |  |  | 0.96 | 0.99 | 2.94 | 0.95 | 0.22 |  |  |  | 1.08 | 0.91 | 2.04 | 1 | 0.2 |
|  |  |  |  |  |  |  |  |  |  |  |  |  |  |  |  |
|  |  |  |  |  |  |  |  |  |  |  |  |  |  |  |  |
|  |  |  |  |  |  |  |  |  |  |  |  |  |  |  |  |
|  |  |  |  |  |  |  |  |  |  |  |  |  |  |  |  |
|  |  |  |  |  |  |  |  |  |  |  |  |  |  |  |  |
|  |  |  |  |  |  |  |  |  |  |  |  |  |  |  |  |
|  |  |  |  |  |  |  |  |  |  |  |  |  |  |  |  |
|  |  |  |  |  |  |  |  |  |  |  |  |  |  |  |  |
|  |  | caspase-3 | Control | pcDNA-NC | pcDNA-TFF3 | si-NC | si-TFF3 |  |  | caspase-3 | Control | pcDNA-NC | pcDNA-TFF3 | si-NC | si-TFF3 |
|  |  |  | 1.1 | 0.9 | 1.7 | 0.95 | 0.48 |  |  |  | 1.06 | 1.1 | 3 | 0.99 | 0.18 |
|  |  |  | 1 | 0.93 | 1.75 | 1.06 | 0.38 |  |  |  | 0.97 | 1.03 | 2.89 | 1.06 | 0.13 |
|  |  |  | 0.89 | 1.06 | 1.97 | 0.92 | 0.5 |  |  |  | 0.9 | 0.89 | 2.71 | 0.92 | 0.12 |
|  |  |  |  |  |  |  |  |  |  |  |  |  |  |  |  |
|  |  |  |  |  |  |  |  |  |  |  |  |  |  |  |  |
|  |  |  |  |  |  |  |  |  |  |  |  |  |  |  |  |
|  |  |  |  |  |  |  |  |  |  |  |  |  |  |  |  |
|  |  |  |  |  |  |  |  |  |  |  |  |  |  |  |  |
|  |  |  |  |  |  |  |  |  |  |  |  |  |  |  |  |
|  |  |  |  |  |  |  |  |  |  |  |  |  |  |  |  |
|  |  |  |  |  |  |  |  |  |  |  |  |  |  |  |  |
|  |  | Bcl-2 | Control | pcDNA-NC | pcDNA-TFF3 | si-NC | si-TFF3 |  |  | Bcl-2 | Control | pcDNA-NC | pcDNA-TFF3 | si-NC | si-TFF3 |
|  |  |  | 1.03 | 0.99 | 0.35 | 0.98 | 1.78 |  |  |  | 1.02 | 1.03 | 0.32 | 1.13 | 2.36 |
|  |  |  | 0.97 | 0.97 | 0.32 | 1.04 | 1.62 |  |  |  | 1.09 | 1.13 | 0.38 | 1.08 | 2.19 |
|  |  |  | 1.14 | 1.06 | 0.26 | 0.94 | 1.54 |  |  |  | 0.92 | 1 | 0.29 | 0.96 | 2.07 |
|  |  |  |  |  |  |  |  |  |  |  |  |  |  |  |  |

**4A**

| transwell-migration |  | BCPAP |  |  |  |  |  |  |  | TPC-1 |  |  |  |  |  |
| --- | --- | --- | --- | --- | --- | --- | --- | --- | --- | --- | --- | --- | --- | --- | --- |
|  |  |  | Control | pcDNA-NC | pcDNA-TFF3 | si-NC | si-TFF3 |  |  |  | Control | pcDNA-NC | pcDNA-TFF3 | si-NC | si-TFF3 |
|  |  |  | 175 | 169 | 93 | 160 | 366 |  |  |  | 75 | 80 | 37 | 82 | 137 |
|  |  |  | 196 | 183 | 106 | 185 | 399 |  |  |  | 88 | 88 | 46 | 89 | 152 |
|  |  |  | 152 | 154 | 80 | 151 | 331 |  |  |  | 63 | 71 | 26 | 74 | 121 |
|  |  |  |  |  |  |  |  |  |  |  |  |  |  |  |  |

**4B**

| transwell-invasion |  | BCPAP |  |  |  |  |  |  |  | TPC-1 |  |  |  |  |  |
| --- | --- | --- | --- | --- | --- | --- | --- | --- | --- | --- | --- | --- | --- | --- | --- |
|  |  |  | Control | pcDNA-NC | pcDNA-TFF3 | si-NC | si-TFF3 |  |  |  | Control | pcDNA-NC | pcDNA-TFF3 | si-NC | si-TFF3 |
|  |  |  | 102 | 105 | 33 | 100 | 246 |  |  |  | 70 | 73 | 30 | 75 | 126 |
|  |  |  | 119 | 117 | 26 | 108 | 271 |  |  |  | 62 | 82 | 26 | 82 | 139 |
|  |  |  | 92 | 96 | 37 | 88 | 225 |  |  |  | 79 | 63 | 35 | 67 | 112 |
|  |  |  |  |  |  |  |  |  |  |  |  |  |  |  |  |
|  |  |  |  |  |  |  |  |  |  |  |  |  |  |  |  |

**4C**

| western blot |  | BCPAP |  |  |  |  |  |  |  | TPC-1 |  |  |  |  |  |  |
| --- | --- | --- | --- | --- | --- | --- | --- | --- | --- | --- | --- | --- | --- | --- | --- | --- |
|  |  | Vimentin | Control | pcDNA-NC | pcDNA-TFF3 | si-NC | si-TFF3 |  |  | Vimentin | Control | pcDNA-NC | pcDNA-TFF3 | si-NC | si-TFF3 |  |
|  |  |  | 1.03 | 1.05 | 0.25 | 0.94 | 1.55 |  |  |  | 0.91 | 1.09 | 0.25 | 1.08 | 1.86 |  |
|  |  |  | 1.07 | 1.02 | 0.29 | 1.1 | 1.42 |  |  |  | 0.98 | 1.01 | 0.29 | 1.03 | 1.7 |  |
|  |  |  | 0.99 | 0.95 | 0.22 | 0.91 | 1.4 |  |  |  | 1.01 | 0.92 | 0.24 | 0.9 | 1.64 |  |
|  |  |  |  |  |  |  |  |  |  |  |  |  |  |  |  |  |
|  |  |  |  |  |  |  |  |  |  |  |  |  |  |  |  |  |
|  |  |  |  |  |  |  |  |  |  |  |  |  |  |  |  |  |
|  |  |  |  |  |  |  |  |  |  |  |  |  |  |  |  |  |
|  |  |  |  |  |  |  |  |  |  |  |  |  |  |  |  |  |
|  |  |  |  |  |  |  |  |  |  |  |  |  |  |  |  |  |
|  |  |  |  |  |  |  |  |  |  |  |  |  |  |  |  |  |
|  |  |  |  |  |  |  |  |  |  |  |  |  |  |  |  |  |
|  |  | N-cadherin | Control | pcDNA-NC | pcDNA-TFF3 | si-NC | si-TFF3 |  |  | N-cadherin | Control | pcDNA-NC | pcDNA-TFF3 | si-NC | si-TFF3 |  |
|  |  |  | 1.07 | 1.08 | 0.42 | 1.09 | 1.97 |  |  |  | 1.08 | 1.11 | 0.4 | 0.97 | 2.01 |  |
|  |  |  | 0.96 | 1.02 | 0.34 | 1.07 | 1.88 |  |  |  | 0.99 | 1.07 | 0.33 | 0.88 | 1.88 |  |
|  |  |  | 0.91 | 0.98 | 0.33 | 1 | 1.78 |  |  |  | 0.93 | 0.95 | 0.31 | 1.04 | 1.75 |  |
|  |  |  |  |  |  |  |  |  |  |  |  |  |  |  |  |  |
|  |  |  |  |  |  |  |  |  |  |  |  |  |  |  |  |  |
|  |  |  |  |  |  |  |  |  |  |  |  |  |  |  |  |  |
|  |  |  |  |  |  |  |  |  |  |  |  |  |  |  |  |  |
|  |  |  |  |  |  |  |  |  |  |  |  |  |  |  |  |  |
|  |  |  |  |  |  |  |  |  |  |  |  |  |  |  |  |  |
|  |  |  |  |  |  |  |  |  |  |  |  |  |  |  |  |  |
|  |  |  |  |  |  |  |  |  |  |  |  |  |  |  |  |  |
|  |  | E-cadherin | Control | pcDNA-NC | pcDNA-TFF3 | si-NC | si-TFF3 |  |  | E-cadherin | Control | pcDNA-NC | pcDNA-TFF3 | si-NC | si-TFF3 |  |
|  |  |  | 1.06 | 0.92 | 2.47 | 1.04 | 0.22 |  |  |  | 1.04 | 1.04 | 1.83 | 0.96 | 0.34 |  |
|  |  |  | 0.95 | 0.96 | 2.35 | 0.92 | 0.2 |  |  |  | 1.02 | 1 | 1.7 | 0.9 | 0.42 |  |
|  |  |  | 1 | 1.08 | 2.19 | 0.93 | 0.18 |  |  |  | 0.97 | 0.95 | 1.63 | 1.03 | 0.4 |  |
|  |  |  |  |  |  |  |  |  |  |  |  |  |  |  |  |  |

**5B**

| western blot |  | BCPAP |  |  |  |  |  |
| --- | --- | --- | --- | --- | --- | --- | --- |
|  |  | IL-6 | Control | pcDNA-NC | pcDNA-TFF3 | si-NC | si-TFF3 |
|  |  |  | 0.98 | 0.92 | 0.46 | 1.1 | 3.99 |
|  |  |  | 1 | 0.99 | 0.4 | 1.1 | 3.83 |
|  |  |  | 1.03 | 1.05 | 0.38 | 0.94 | 3.54 |
|  |  |  |  |  |  |  |  |
|  |  |  |  |  |  |  |  |
|  |  |  |  |  |  |  |  |
|  |  |  |  |  |  |  |  |
|  |  |  |  |  |  |  |  |
|  |  |  |  |  |  |  |  |
|  |  |  |  |  |  |  |  |
|  |  |  |  |  |  |  |  |
|  |  | p-JAK2/JAK2 | Control | pcDNA-NC | pcDNA-TFF3 | si-NC | si-TFF3 |
|  |  |  | 1.02 | 1 | 0.23 | 1.07 | 2.86 |
|  |  |  | 0.99 | 0.94 | 0.19 | 1 | 2.74 |
|  |  |  | 1.14 | 1.04 | 0.27 | 0.94 | 2.53 |
|  |  |  |  |  |  |  |  |
|  |  |  |  |  |  |  |  |
|  |  |  |  |  |  |  |  |
|  |  |  |  |  |  |  |  |
|  |  |  |  |  |  |  |  |
|  |  |  |  |  |  |  |  |
|  |  |  |  |  |  |  |  |
|  |  |  |  |  |  |  |  |
|  |  | p-STAT3/STAT3 | Control | pcDNA-NC | pcDNA-TFF3 | si-NC | si-TFF3 |
|  |  |  | 1.03 | 1.07 | 0.34 | 1.09 | 3.5 |
|  |  |  | 1.1 | 0.95 | 0.32 | 1 | 3.37 |
|  |  |  | 0.9 | 1.06 | 0.29 | 0.92 | 3.12 |
|  |  |  |  |  |  |  |  |

**5C**

| western blot |  | TPC-1 |  |  |  |  |  |
| --- | --- | --- | --- | --- | --- | --- | --- |
|  |  | IL-6 | Control | pcDNA-NC | pcDNA-TFF3 | si-NC | si-TFF3 |
|  |  |  | 0.93 | 1.1 | 0.29 | 1.18 | 3.35 |
|  |  |  | 0.97 | 1 | 0.32 | 1.1 | 3.1 |
|  |  |  | 1.08 | 0.97 | 0.27 | 1.01 | 2.95 |
|  |  |  |  |  |  |  |  |
|  |  |  |  |  |  |  |  |
|  |  |  |  |  |  |  |  |
|  |  |  |  |  |  |  |  |
|  |  |  |  |  |  |  |  |
|  |  |  |  |  |  |  |  |
|  |  |  |  |  |  |  |  |
|  |  |  |  |  |  |  |  |
|  |  | p-JAK2/JAK2 | Control | pcDNA-NC | pcDNA-TFF3 | si-NC | si-TFF3 |
|  |  |  | 1.09 | 0.98 | 0.39 | 1 | 1.89 |
|  |  |  | 1.08 | 1.06 | 0.29 | 1.08 | 1.77 |
|  |  |  | 0.92 | 0.91 | 0.31 | 0.91 | 1.63 |
|  |  |  |  |  |  |  |  |
|  |  |  |  |  |  |  |  |
|  |  |  |  |  |  |  |  |
|  |  |  |  |  |  |  |  |
|  |  |  |  |  |  |  |  |
|  |  |  |  |  |  |  |  |
|  |  |  |  |  |  |  |  |
|  |  |  |  |  |  |  |  |
|  |  | p-STAT3/STAT3 | Control | pcDNA-NC | pcDNA-TFF3 | si-NC | si-TFF3 |
|  |  |  | 1.1 | 0.95 | 0.11 | 0.93 | 2.22 |
|  |  |  | 1.1 | 1.06 | 0.12 | 1.03 | 2.38 |
|  |  |  | 0.94 | 0.9 | 0.08 | 0.92 | 2.05 |
|  |  |  |  |  |  |  |  |
