# Supplementary material for: Trefoil Factor 3 Inhibits Thyroid Cancer Cell Progression Related to IL-6/JAK/STAT3 Signaling Pathway
Source: Evid Based Complement Alternat Med. 2021 Sep 14;2021:2130229. doi: 10.1155/2021/2130229 (PMC8457945; doi:10.1155/2021/2130229)
Supplement: Supplementary Materials — The original experimental data of the manuscript are included in the supplementary files named “original data” and “original files.” [file 2130229.f1.zip › 2130229.f1/original files.docx]

Fig 1F











Fig 2B

BCPAP











TPC-1











Fig 2C

BCPAP


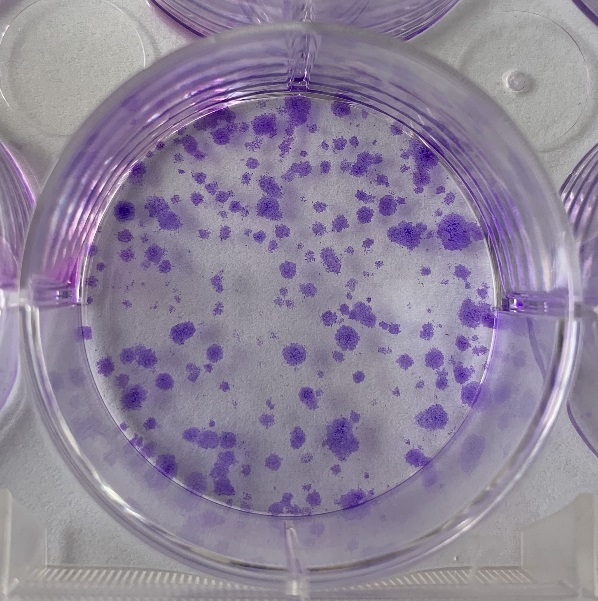

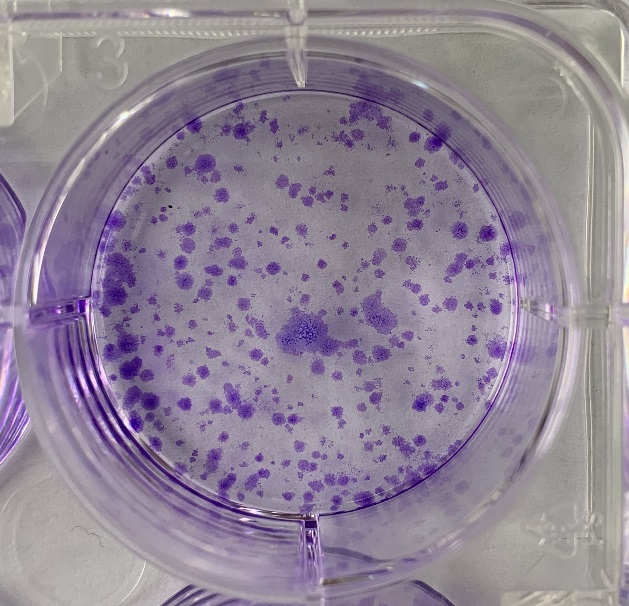


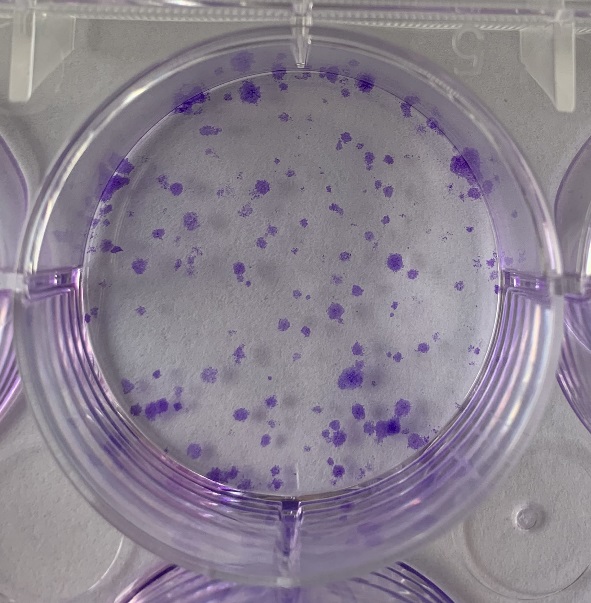

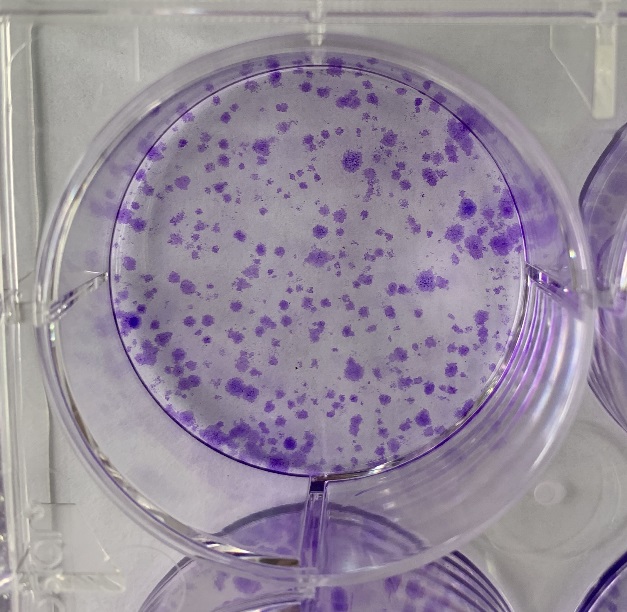

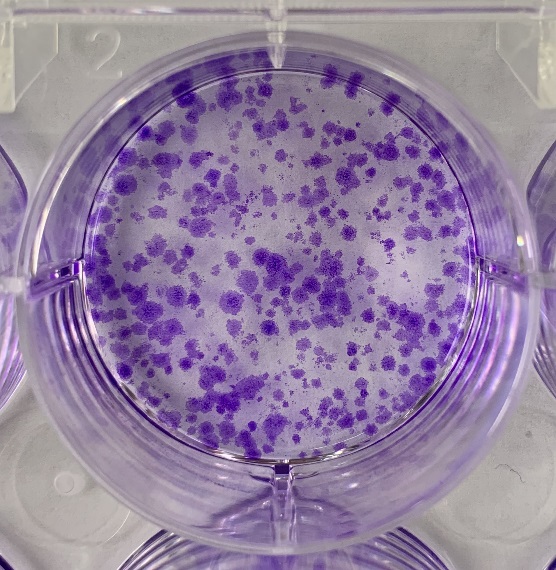


TPC-1


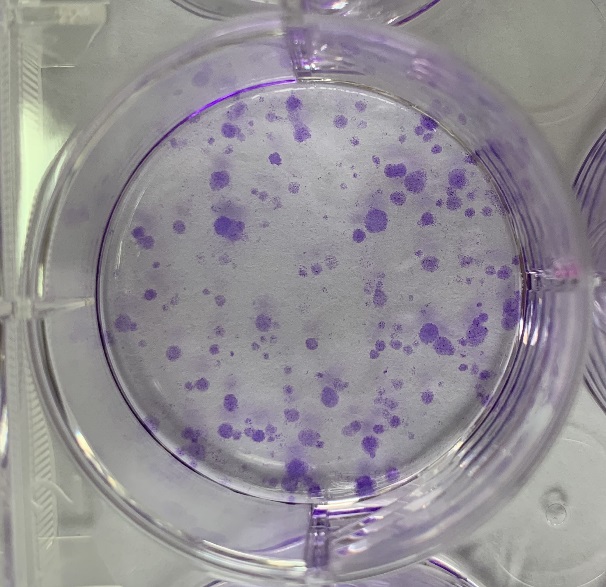

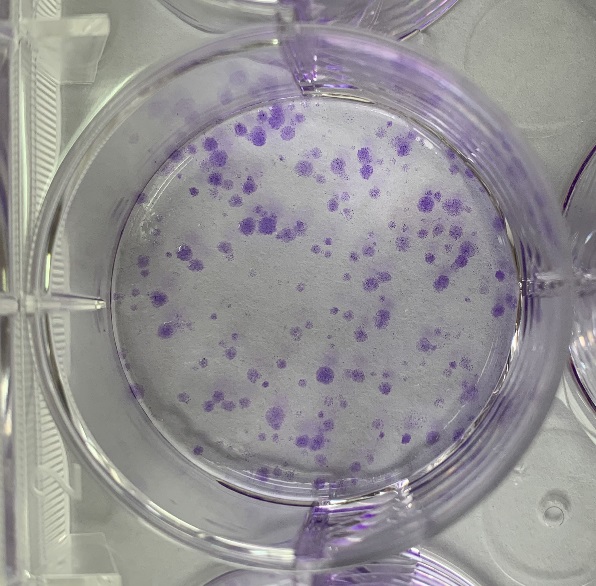

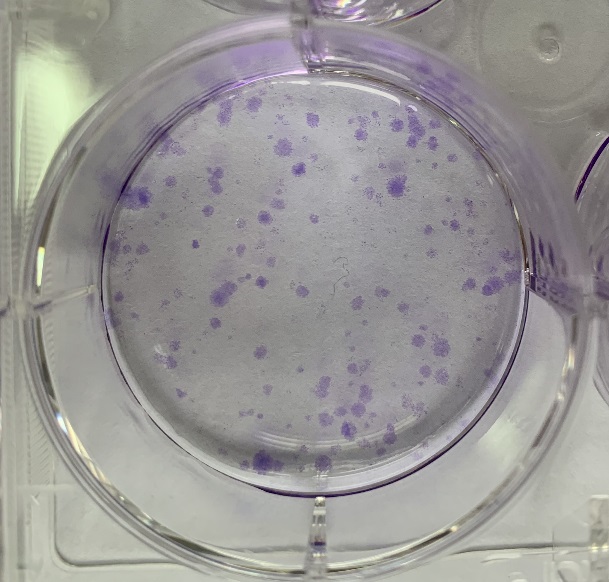

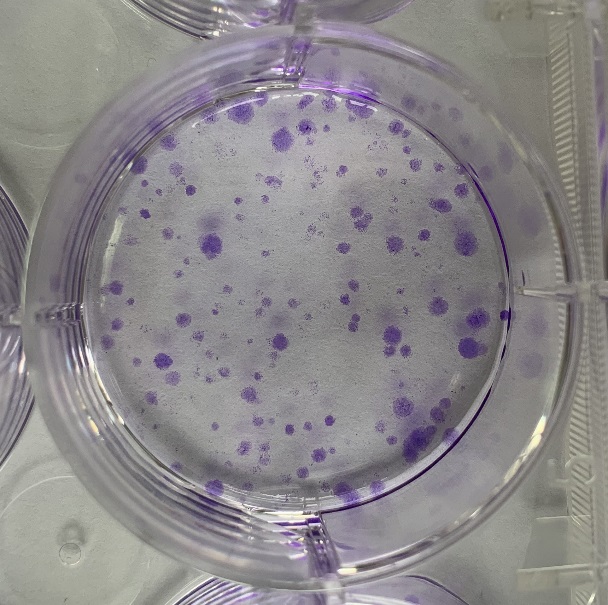

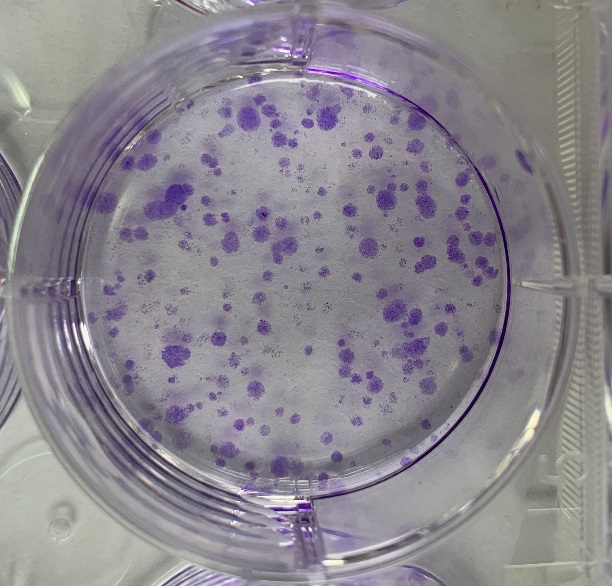


Fig 2D

BCPAP


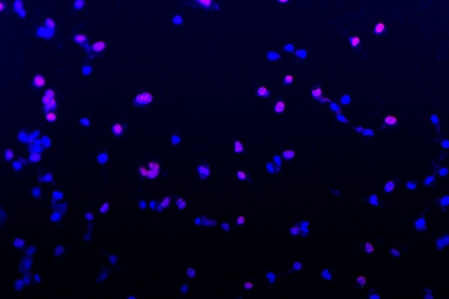

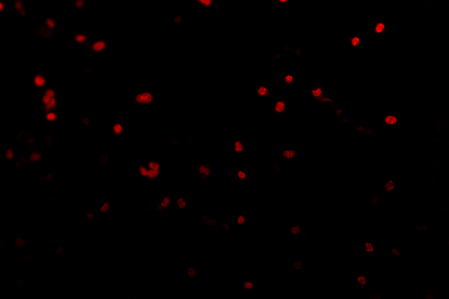

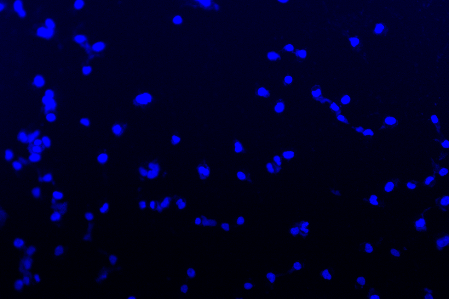


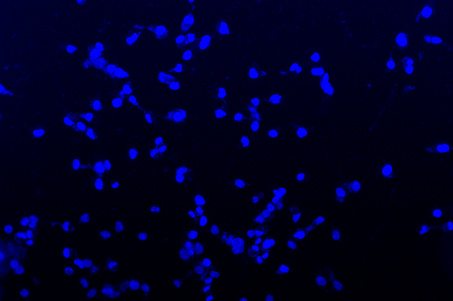


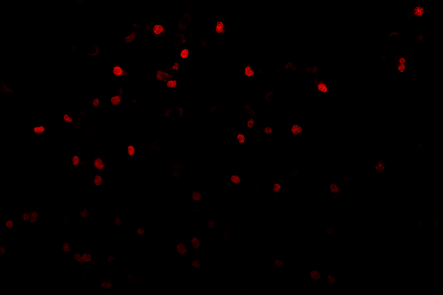

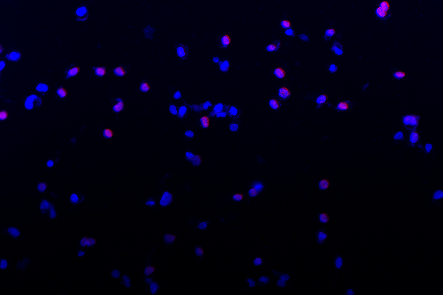

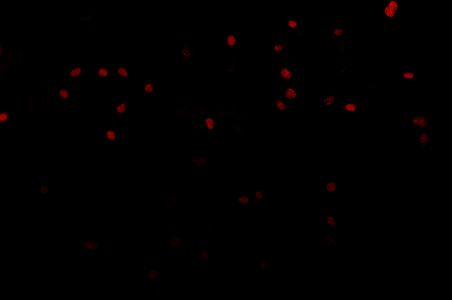

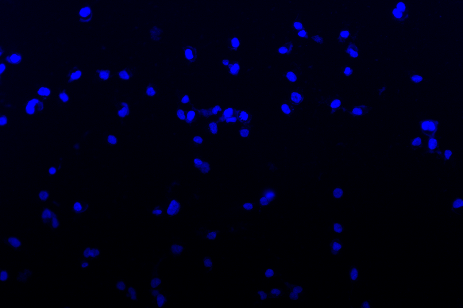

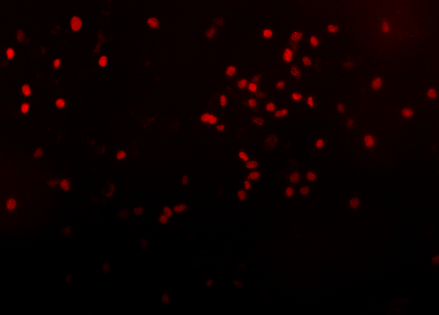

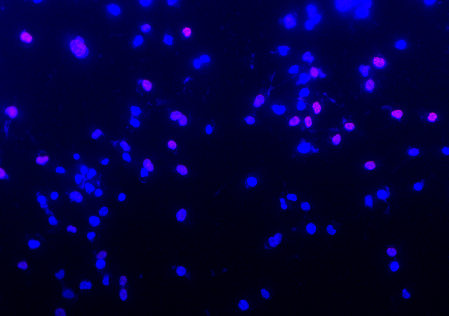

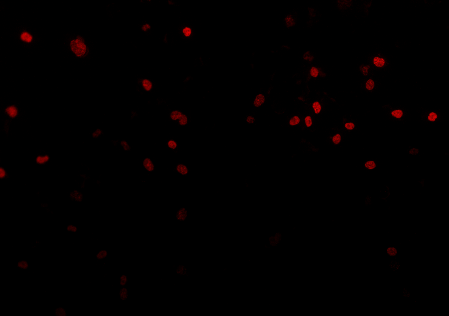

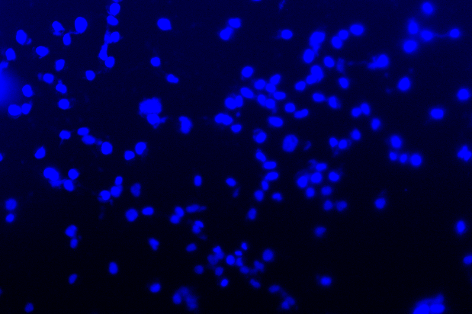

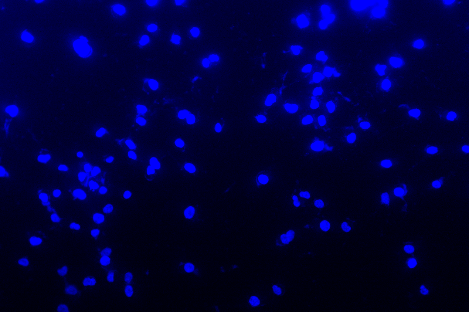

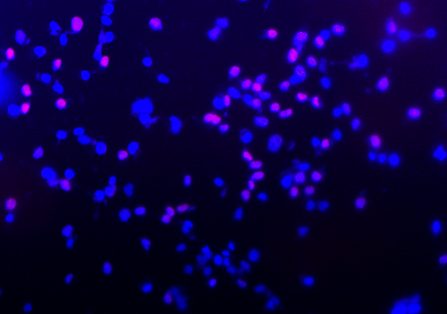

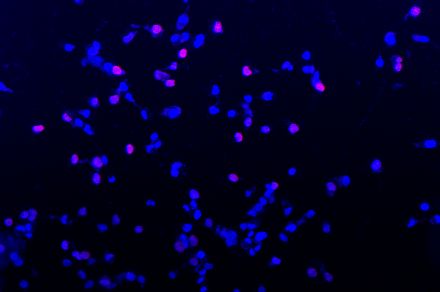


TPC-1


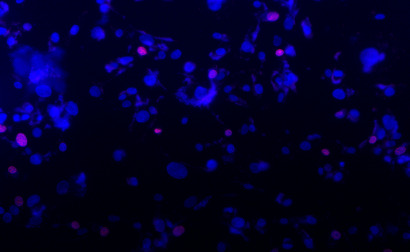

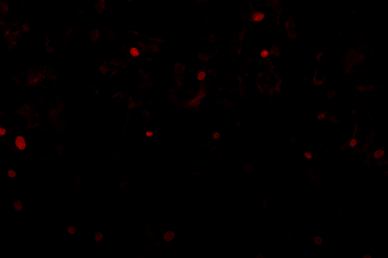

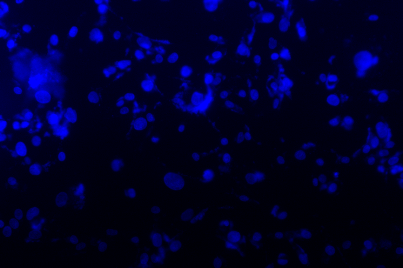

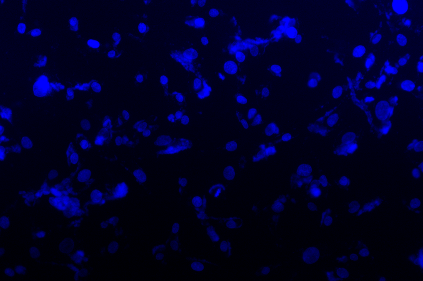


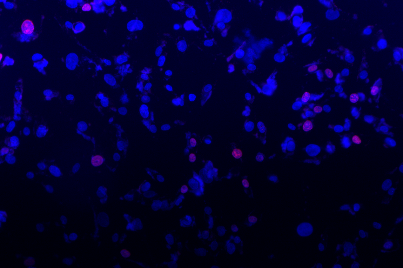

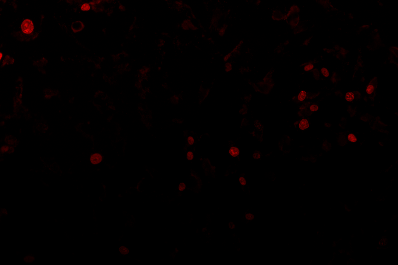

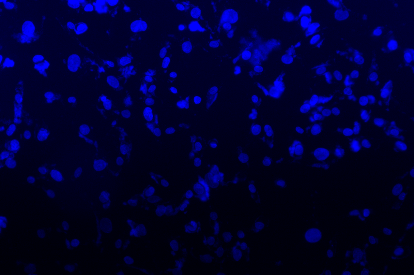


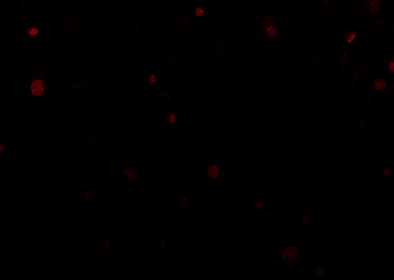

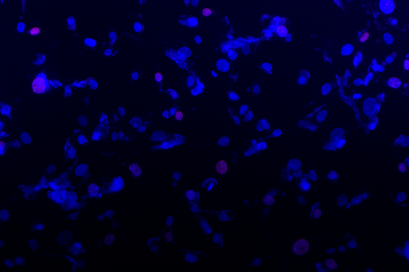


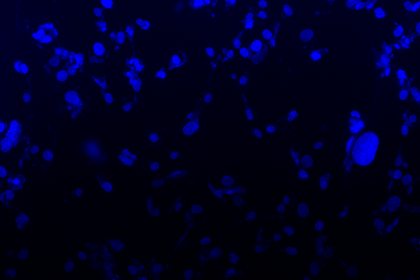

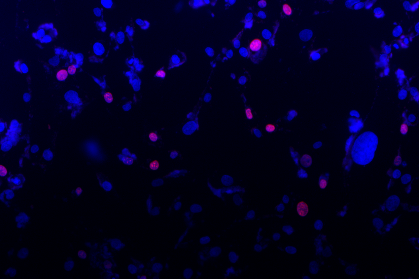

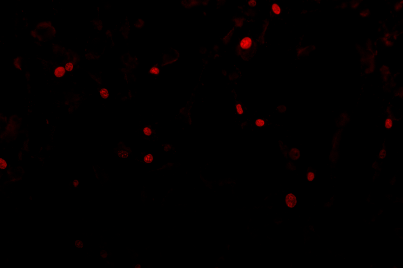


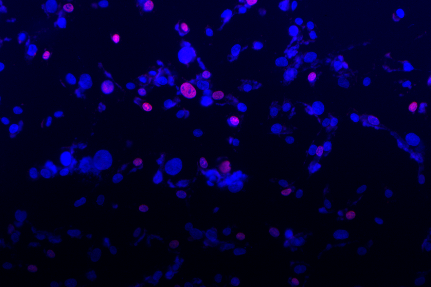

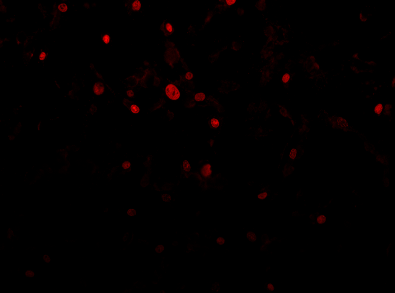

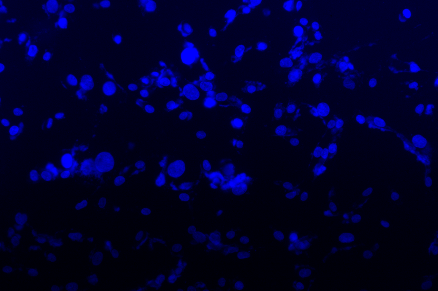


Fig 3A

BCPAP


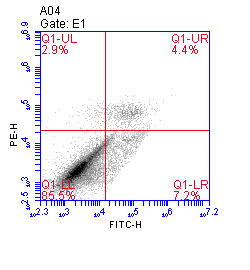

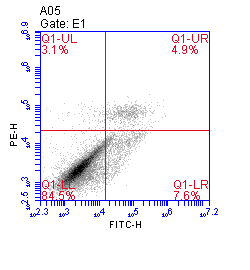

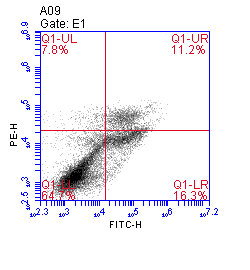

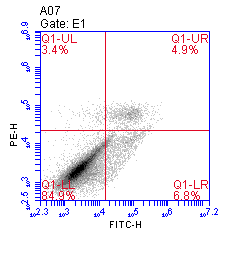

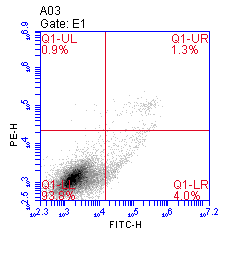


TPC-1


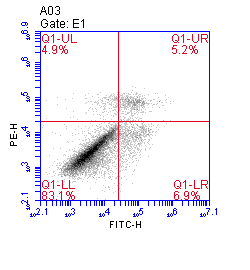

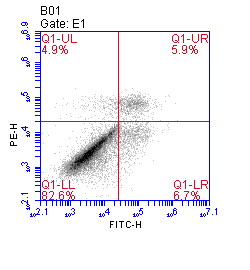

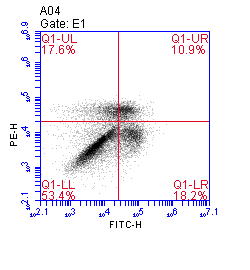

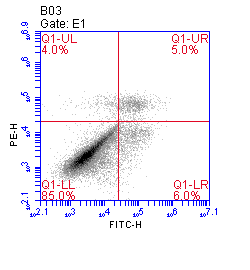

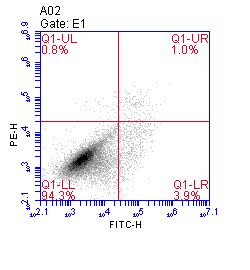


Fig 3B

BCPAP



















TPC-1



















Fig 4A

BCPAP


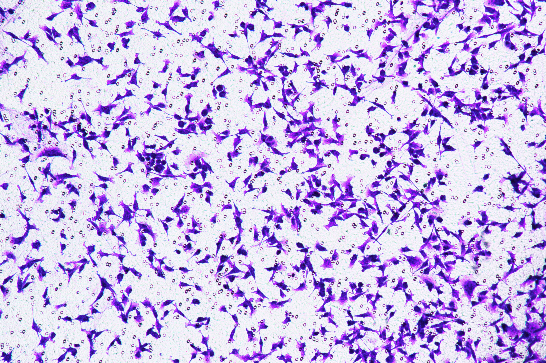

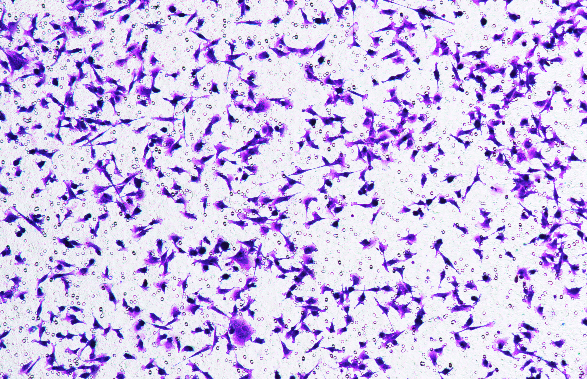

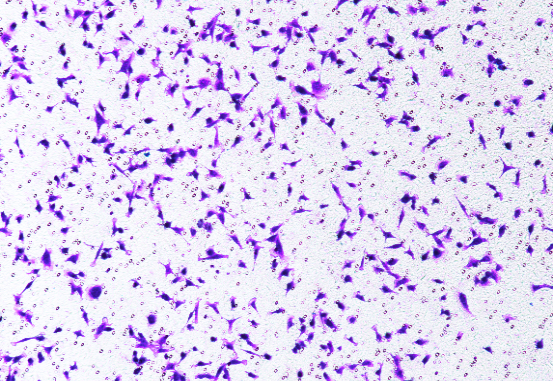

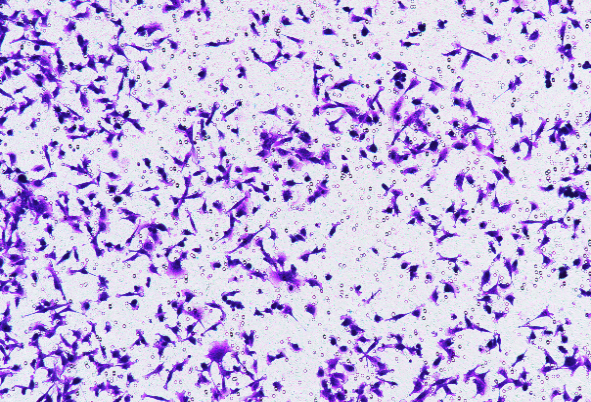

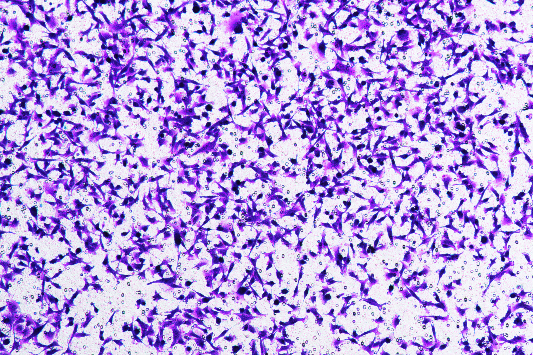


TPC-1


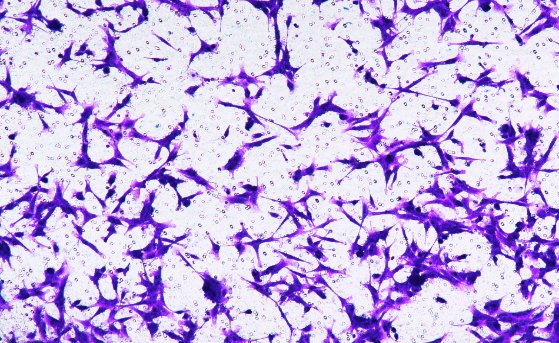

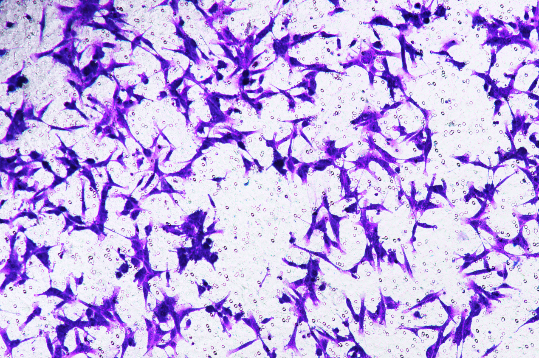

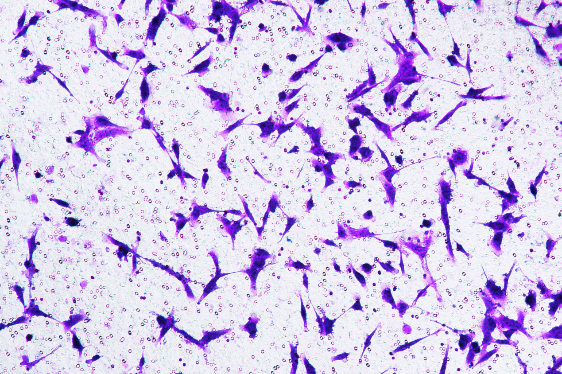

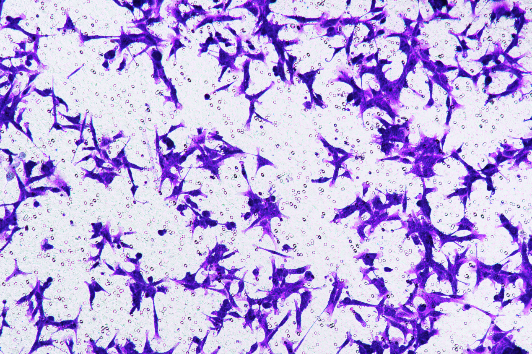

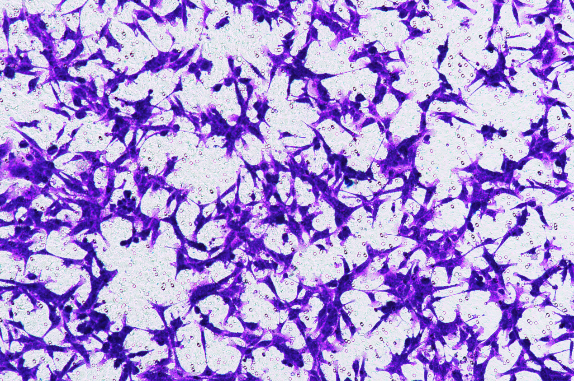


Fig 4B

BCPAP


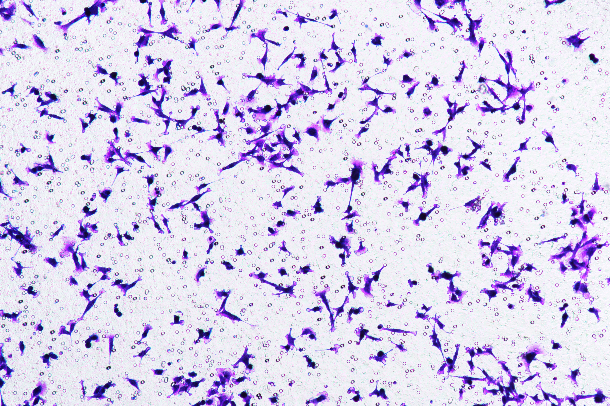

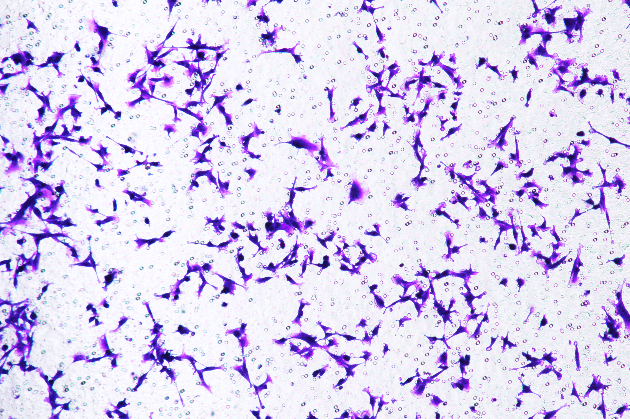

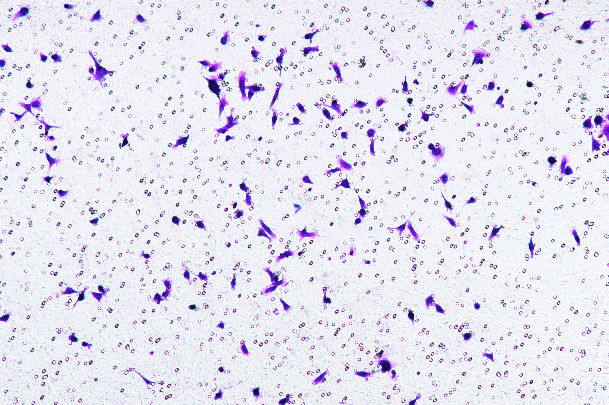

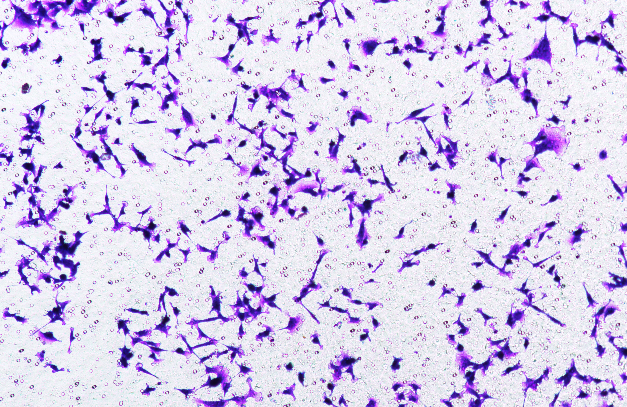

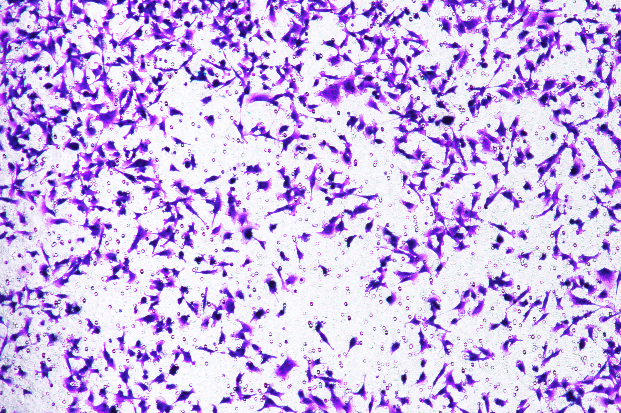


TPC-1


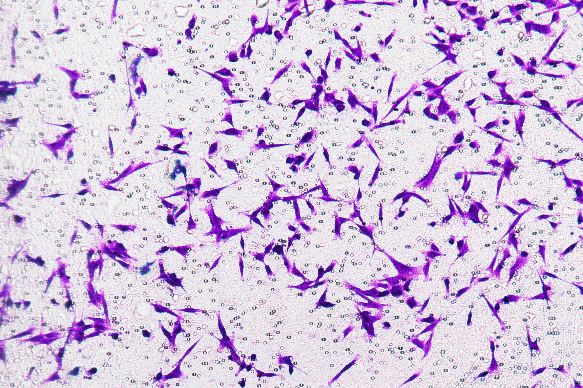

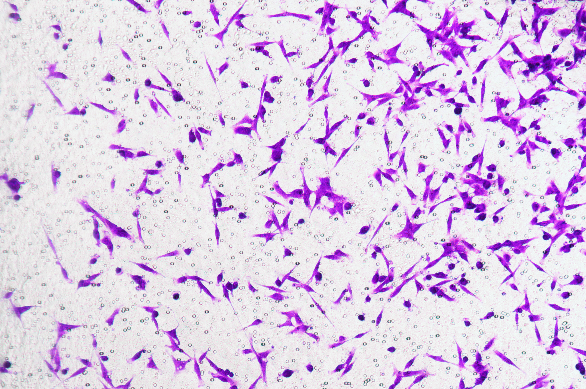

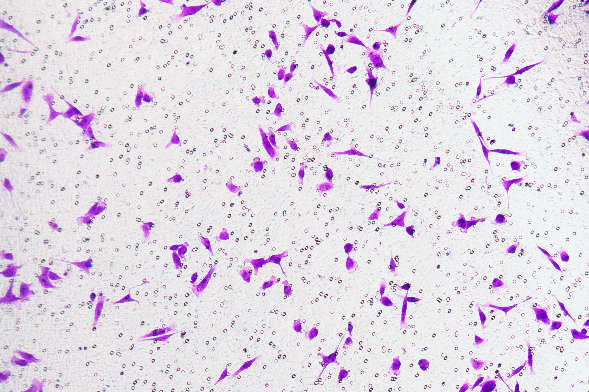

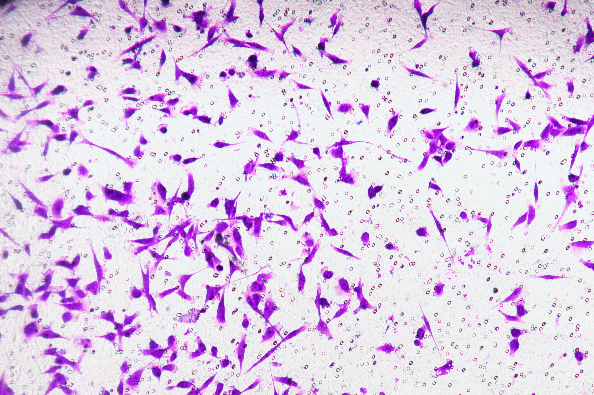

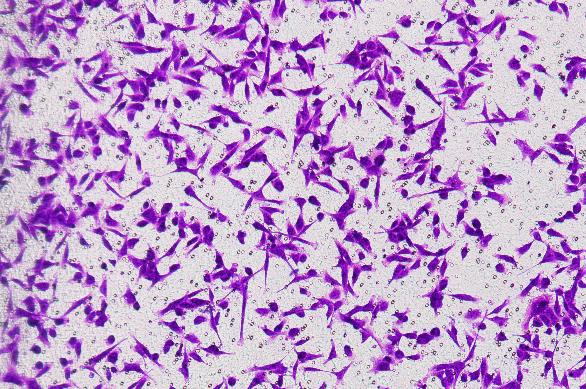


Fig 4C

BCPAP







TPC-1

Fig 5B

Fig 5C
