# Supplementary material for: Trefoil Factor 3 Inhibits Thyroid Cancer Cell Progression Related to IL-6/JAK/STAT3 Signaling Pathway
Source: Evid Based Complement Alternat Med. 2021 Sep 14;2021:2130229. doi: 10.1155/2021/2130229 (PMC8457945; doi:10.1155/2021/2130229)
Supplement: Supplementary Materials — The original experimental data of the manuscript are included in the supplementary files named “original data” and “original files.” [file 2130229.f1.zip › 2130229.f1/Response to editor.docx]

Dear editor,

Thank you for your letter. The supplementary files named “original data” and “original files” which are submitted last time  is the original experimental data submitted at the request of reviewer 2. They are for reviewer 2 to review. Therefore, there is no need for text citation and supplementary description to add into the article.

If there are any other questions, please contact us with no hesitate.

Best wishes

Xiaoling Shang
